# Microbial Diversity and Sulfur Cycling in an Early Earth Analogue: From Ancient Novelty to Modern Commonality

**DOI:** 10.1128/mbio.00016-22

**Published:** 2022-03-08

**Authors:** C. Ryan Hahn, Ibrahim F. Farag, Chelsea L. Murphy, Mircea Podar, Mostafa S. Elshahed, Noha H. Youssef

**Affiliations:** a Department of Microbiology and Molecular Genetics, Oklahoma State University, Stillwater, Oklahoma, USA; b Department of Microbiology, University of Tennessee Knoxville, Knoxville, Tennessee, USA; c Oak Ridge National Laboratory, Oak Ridge, Tennessee, USA; Univ. of Vienna; University of Vienna

**Keywords:** genome-resolved metagenomics, sulfur cycling, evolution, preoxygenated earth

## Abstract

Life emerged and diversified in the absence of molecular oxygen. The prevailing anoxia and unique sulfur chemistry in the Paleo-, Meso-, and Neoarchean and early Proterozoic eras may have supported microbial communities that differ from those currently thriving on the earth’s surface. Zodletone spring in southwestern Oklahoma represents a unique habitat where spatial sampling could substitute for geological eras namely, from the anoxic, surficial light-exposed sediments simulating a preoxygenated earth to overlaid water column where air exposure simulates oxygen intrusion during the Neoproterozoic era. We document a remarkably diverse microbial community in the anoxic spring sediments, with 340/516 (65.89%) of genomes recovered in a metagenomic survey belonging to 200 bacterial and archaeal families that were either previously undescribed or that exhibit an extremely rare distribution on the current earth. Such diversity is underpinned by the widespread occurrence of sulfite, thiosulfate, tetrathionate, and sulfur reduction and the paucity of sulfate reduction machineries in these taxa. Hence, these processes greatly expand lineages mediating reductive sulfur-cycling processes in the tree of life. An analysis of the overlaying oxygenated water community demonstrated the development of a significantly less diverse community dominated by well-characterized lineages and a prevalence of oxidative sulfur-cycling processes. Such a transition from ancient novelty to modern commonality underscores the profound impact of the great oxygenation event on the earth’s surficial anoxic community. It also suggests that novel and rare lineages encountered in current anaerobic habitats could represent taxa that once thrived in an anoxic earth but have failed to adapt to earth’s progressive oxygenation.

## INTRODUCTION

Sulfur is one of the most abundant elements on earth, exhibiting a wide range of oxidation states (−2 to +6). Microorganisms have evolved a plethora of genes and pathways for exploiting sulfur-redox reactions for energy generation. Reductive processes employ sulfur oxyanions or elemental sulfur as terminal electron acceptors in anaerobic respiratory schemes linked to heterotrophic or autotrophic growth. Oxidative processes, on the other hand, employ sulfides or elemental sulfur as electron donors, powering chemolithotrophic and photosynthetic growth.

Thermodynamic considerations limit reductive sulfur processes to habitats where oxygen is limited. This habitat restriction is reflected in the global distribution of microorganisms that reduce sulfate (SO_4_^2−^), sulfite (SO_3_^2−^), thiosulfate (S_2_O_3_^2−^), tetrathionate (S_4_O_6_^2−^), and elemental sulfur (S^0^) (henceforth collectively referred to as SRM) in permanently and seasonally anoxic and hypoxic habitats in marine (1–3), freshwater (4), terrestrial (5), and subsurface (6) ecosystems. Sulfate is highly abundant on the current earth. Hence, sulfate reduction dominates reductive processes in the global sulfur cycle, although the reduction and disproportionation of the intermediate sulfur species, e.g., sulfur (7, 8), sulfite (8), thiosulfate, and tetrathionate (9, 10), could be significant in localized settings.

The history of earth’s sulfur cycle is a prime example of a geological-biological feedback loop, where the evolution of biological processes is driven by, and dramatically impacts, the earth’s biogeochemistry. The earth’s surface was completely anoxic during the first two billion years of its history, and the availability and speciation of various sulfur species differed greatly from their current values. Sulfate levels were significantly lower than to current values in oceanic water (28 mM), with estimates of <200 μM to 1mM from the Archean up to the Paleoproterozoic (2.3 gigayears ago [Gya]) eras (11–14). On the other hand, intermediate sulfur species appear to have played an important role in shaping the ancient sulfur cycle (15). Modeling suggests that mM levels of SO_3_^2−^ were attained in the Archean anoxic shallow surficial aquifers as a result of the dissolution of the volcanic SO_2_ prevailing in aquatic habitats (12). Isotopic studies have demonstrated the importance of elemental sulfur, sulfite, and thiosulfate reduction in the Archean era (15, 16).

The evolution of life (3.8 to 4.0 Gya) in the early Archean era and the subsequent evolution of major bacterial and archaeal clades in the late Archean and early Proterozoic eras (17) occurred within this background of anoxia and characteristic sulfur chemistry. As such, it has been speculated that organisms using intermediate forms of sulfur were likely more common than sulfate-reducing organisms (15). However, while isotopic fractionation, modeling, and microscopic studies could provide clues on prevailing sulfur speciation patterns and prevalent biological processes, the identity of microorganisms mediating such processes is unknown. This knowledge gap is due mostly to constrains on the preservation of nucleic acids and other biological macromolecules, with the oldest successful DNA-sequenced sample being only 1.2 million years old (18).

Investigation of the microbial community in modern ecosystems with conditions resembling those prevailing in the ancient earth could provide important clues to the nature and identity of microorganisms that thrived under conditions prevailing prior to earth’s oxygenation. In Zodletone spring, a surficial anoxic spring in southwestern Oklahoma, anoxic, surficial, light-exposed conditions are maintained in the sediments by the constant emergence of sulfide-saturated water at the spring source from anoxic underground water formations in the Anadarko Basin, along with gaseous hydrocarbons, which occur in seeps in the general vicinity. These surficial anoxic conditions also support a sulfur chemistry characterized by high levels of sulfide, sulfite, sulfur (soluble polysulfide), thiosulfate, and a low level of sulfate, as reported previously (19–21). Microbial diversity using 16S rRNA amplicon surveys have reported a higher level of phylogenetic diversity in the anoxic spring sediments and the affiliation of a fraction of the spring community with previously recognized sulfur-metabolizing lineages, as well as the high proportion of phylogenetically novel taxa in the spring anoxic sediments (20, 22, 23). As such, the prevailing conditions at the spring source are reminiscent of ancient metabolic capacities prevailing on the earth’s surface in the late Archean/early Proterozoic eras as noted previously (19).

Furthermore, the sediments at the source of the spring are overlaid by an air-exposed water column, and prior microsensor measurements and detailed geochemical analysis (19, 21) demonstrated that oxygen intrusion leads to a vertical oxygen gradient (from oxic in the top 1 μm, to hypoxic in the middle, to anoxic in deeper layers overlaying the sediments) (see Fig. S1 in the supplemental material). As such, contrasting communities between the anoxic sediments and the oxygen-exposed water column could provide a glimpse into how oxygen evolution has altered such communities. Here, we combined metagenomic, metatranscriptomic, and amplicon-based approaches to fully characterize the microbial community in Zodletone spring. Our results provide a glimpse of the community mediating the ancient sulfur cycle, significantly expand the overall microbial diversity by the description of a wide range of novel lineages, and greatly increase the number of lineages documented to mediate reductive sulfur processes in the microbial tree of life.

10.1128/mbio.00016-22.2 FIG S1 Zodletone spring source sediments and overlaid water. Download FIG S1, PDF file, 0.1 MB. Copyright © 2022 Hahn et al. 2022 Hahn et al. https://creativecommons.org/licenses/by/4.0/ This content is distributed under the terms of the Creative Commons Attribution 4.0 International license.

## RESULTS

### Novel phylogenetic diversity in Zodletone sediments.

**FIG 1:**
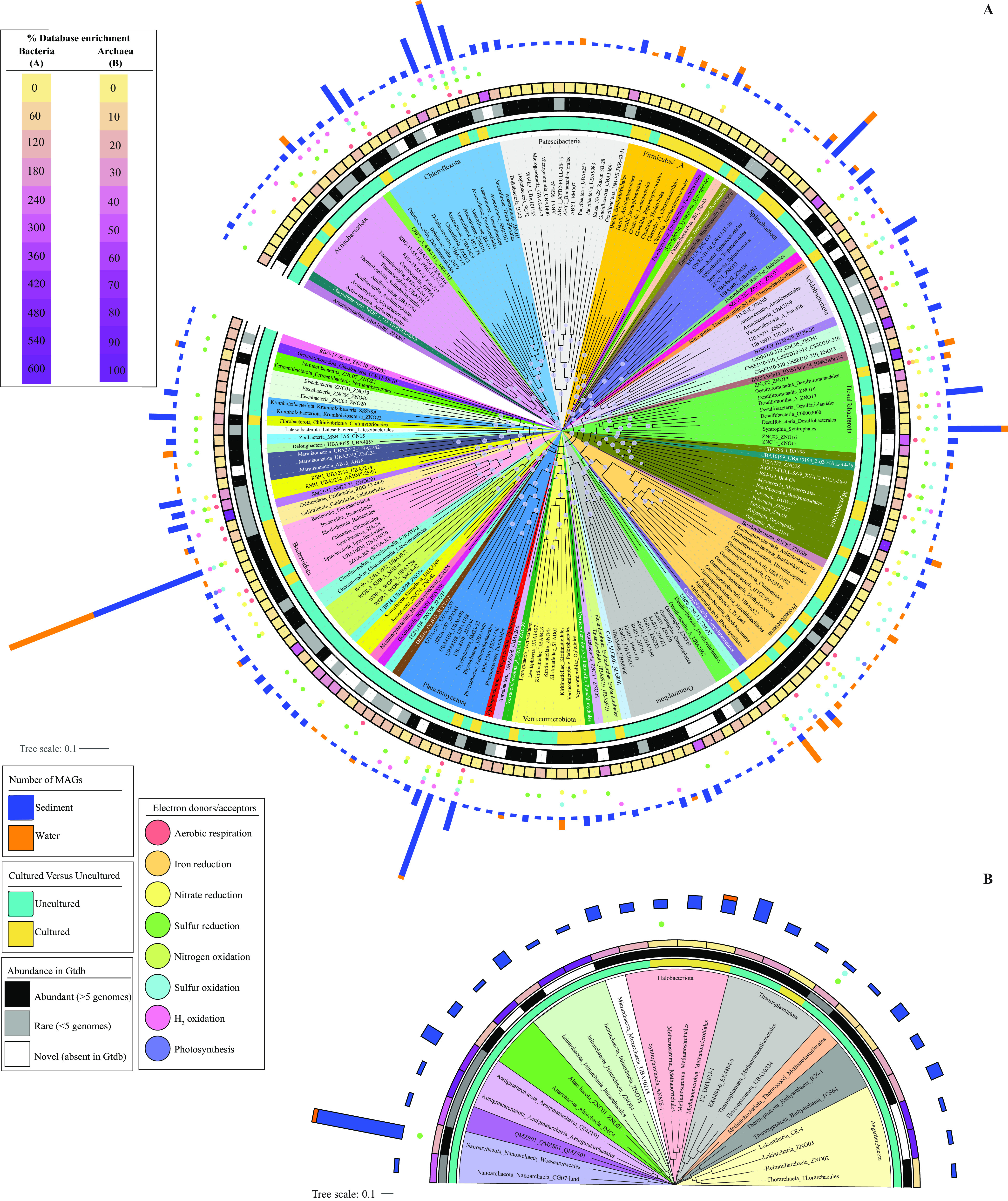
Phylogenomics of the 516 bacterial (A) and 114 archaeal (B) genomes analyzed in this study. The maximum likelihood trees were constructed in FastTree (86) based on the concatenated alignments of 120 (bacterial), and 122 (archaeal) housekeeping genes obtained from GTDB-TK (85). The branches represent order-level taxonomy and are color coded by phylum. For phyla with 4 orders or less, branches are labeled as Phylum_Class_Order. For phyla with more than 4 orders, the phylum is shown at the base of the colored wedge and the branches are labeled as Class_Order. Lineages staring with ZN depict novel lineages (ZNC, novel class; ZNO, novel order). Bootstrap support values are shown as bubbles for nodes with >70% support. Tracks around the tree represent (from innermost to outermost)the following: cultured status at the order level (cultured versus uncultured), abundance in GTDB based on the number of available genomes (abundant with more than 5 genomes, rare with 5 genomes or less, and novel with no genomes in GTDB), percentage database enrichment (calculated as number of genomes belonging to a certain order binned in the current study as a percentage of the number of genomes belonging to the same order in GTDB), energy conservation capabilities depicted by colored circles (salmon, aerobic respiration; orange, Fe^3+^ respiration; yellow, nitrate/nitrite reduction; dark green, reductive sulfur processes; lime green, nitrogen oxidation; cyan, oxidative sulfur-processes; pink, respiratory hydrogen oxidation; and purple, photosynthesis), and the number of MAGs belonging to each order binned from the sediment (blue bars) and the water (orange bars). For orders with 20 or more genomes, the family-level delineation is shown in Fig. 3. These orders are *Anaerolineales* (Fig. 3A), *Bacteroidales* (Fig. 3B), *Sedimentisphaerales* (Fig. 3C), *Spirochaetales* (Fig. 3D), *Syntrophales* (Fig. 3E), and *Woesearchaeales* (Fig. 3F).

Metagenomic sequencing of the spring sediments yielded 281 Gbp, of which 79.54% assembled into 12-Gbp contigs, with 6.8-Gbp contigs longer than 1 Kbp. A total of 1,848 genomes were binned, but only 683 passed quality-control criteria, and 516 remained after dereplication (see Table S1 in the supplemental material). These metagenome-assembled genomes (MAGs) represented 64 phyla or candidate phyla (53 bacterial and 11 archaeal), 127 classes, 198 orders, and 300 families (Fig. 1A and B). A diversity assessment utilizing small subunit ribosomal protein S3 from assembled contigs (*n* = 2,079), as well as a complementary 16S rRNA Illumina sequencing effort (*n* = 309,074 amplicons), identified a higher number of taxa (82 phyla and 1,679 species in the ribosomal protein S3 data set, and 69 phyla and 1,050 species in 16S rRNA data set) (see Fig. S2 in the supplemental material). Nevertheless, the overall community composition profiles generated from all three approaches were broadly similar (Fig. S2), suggesting that the MAG list largely reflects the sediment microbial community.

10.1128/mbio.00016-22.3 FIG S2 Zodletone spring phylum-level community composition based on ribosomal protein S3 (RP-S3) and binned genomes (MAGs), as well as the gene for 16S rRNA for both the sediment and the water samples. Download FIG S2, PDF file, 0.04 MB. Copyright © 2022 Hahn et al. 2022 Hahn et al. https://creativecommons.org/licenses/by/4.0/ This content is distributed under the terms of the Creative Commons Attribution 4.0 International license.

10.1128/mbio.00016-22.8 TABLE S1 List of all genomes analyzed in this study with their NCBI assembly accession numbers, taxonomic classification, sequencing statistics, and general genomic features. Download Table S1, XLSX file, 0.1 MB. Copyright © 2022 Hahn et al. 2022 Hahn et al. https://creativecommons.org/licenses/by/4.0/ This content is distributed under the terms of the Creative Commons Attribution 4.0 International license.

**FIG 2:**
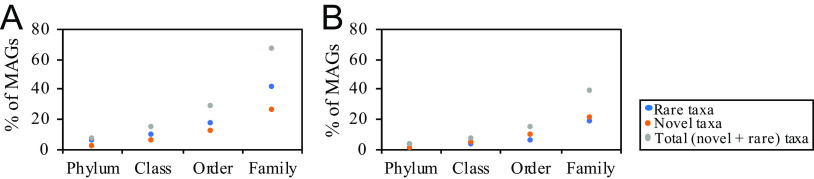
Novelty, rarity, and phylum-level makeup in Zodletone sediment and water communities. Genomes belonging to novel (orange), and LRD (blue) lineages are shown as a percentage of total binned genomes in the sediment (A) and the water (B) communities. The sum of novel and LRD genome percentages is shown in gray.

**FIG 3:**
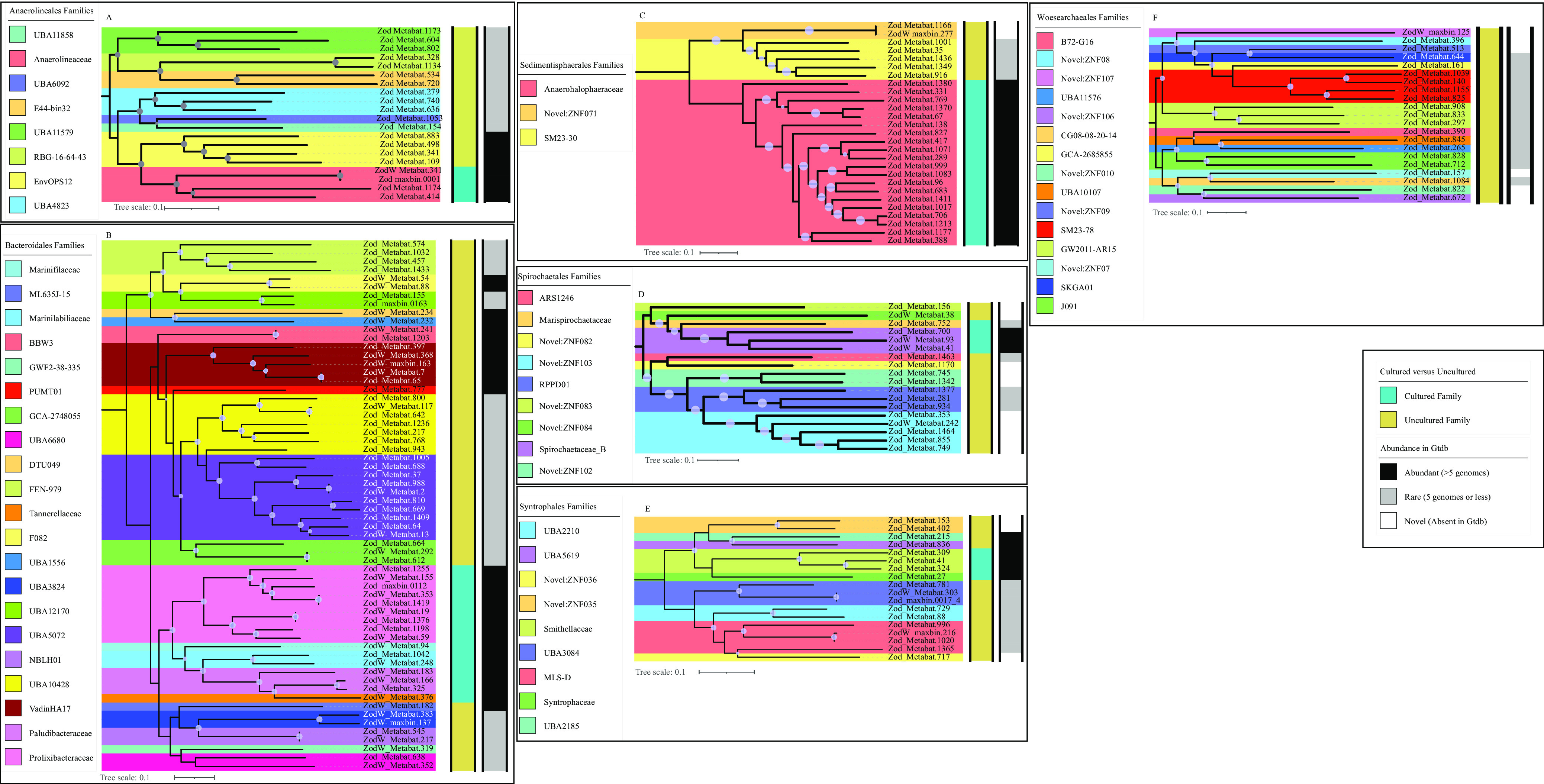
Family-level delineation for orders with 20 or more genomes. The maximum likelihood trees were constructed in FastTree (86) based on the concatenated alignments of 120 and 122 single-copy genes obtained from GTDB-TK (85). Bootstrap support values are shown as bubbles for nodes with >70% support. Families are color coded. To the right of the trees, tracks are shown for cultured status at the family level (cultured versus uncultured) and abundance in GTDB based on the number of available genomes (abundant with more than 5 genomes, rare with 5 genomes or less, and novel with no genomes in GTDB).

An assessment of the novelty and degree of uniqueness of sediment MAGs identified a remarkably high number of previously undescribed lineages (1 phylum, 14 classes, 43 orders, and 97 families) as well as lineages exhibiting rare global distribution (LRD) pattern (11 phyla, 24 classes, 45 orders, and 113 families) in the spring (Fig. 1 to 3). We define LRD lineages as those represented by 5 genomes or less in the Genome Taxonomy Database release 95 (GTDB r95). At the family level, 132 (25.58%) and 208 (40.03%) genomes clustered into 97 novel and 113 LRD families, respectively, bringing the proportion of genomes belonging to novel or LRD families in Zodletone sediments to 65.89%. The high level of novelty in the sediment MAGs is reflected in an average relative evolutionary divergence (RED) value of 0.76, which is a value that is slightly lower than the median RED value for the designation of a novel family (0.77) (24).

The *Chloroflexota* (*n* = 69), *Planctomycetota* (*n* = 47), *Bacteroidota* (*n* = 43), *Desulfobacterota* (*n* = 43), *Spirochaetota* (*n* = 28 genomes), *Patescibacteria* (*n* = 20 genomes), and the archaeal phylum *Nanoarchaeota* (*n* = 21) were the most abundant phyla in Zodletone spring sediments, albeit representing only 52.52% of the total number of recovered genomes (see Text S1 in the supplemental material; Fig. 1 and 3; see Fig. S3 in the supplemental material). An extreme paucity of genomes belonging to the *Proteobacteria* (6 genomes) and *Firmicutes* (12 genomes), which are widely distributed and abundant taxa in current biomes (25), and the absence of oxygen-generating *Cyanobacteria* (0 genomes) were observed (Fig. 1, Fig. S3). Therefore, in addition to expanding the number of novel lineages (classes, orders, and families) and greatly enriching available genomes in rare, poorly represented taxa, our results highlight the uniqueness and distinction of the microbial community thriving in Zodletone spring sediments, compared with those of present earth environments studied so far.

10.1128/mbio.00016-22.1 TEXT S1 Methods for analysis of sulfur-cycling genes are detailed. Details on phylogenomic analysis of Zodletone spring sediments and water communities, the reductive sulfur processes dominating Zodletone spring sediment communities, the oxidative sulfur processes dominating Zodletone water community, the transcriptomic analysis, and additional metabolic capacities in Zodletone spring sediments are also provided. Download Text S1, DOCX file, 0.1 MB. Copyright © 2022 Hahn et al. 2022 Hahn et al. https://creativecommons.org/licenses/by/4.0/ This content is distributed under the terms of the Creative Commons Attribution 4.0 International license.

10.1128/mbio.00016-22.4 FIG S3 Phylum-level affiliation for sediment versus water genomes. Number of genomes belonging to each phylum is shown for the sediment (blue bars on the left) and the water (orange bars on the right). Download FIG S3, PDF file, 0.2 MB. Copyright © 2022 Hahn et al. 2022 Hahn et al. https://creativecommons.org/licenses/by/4.0/ This content is distributed under the terms of the Creative Commons Attribution 4.0 International license.

### Oxygen intrusion reduces the proportion of novel and rare lineages in Zodletone spring.

Metagenomic sequencing of the oxygen-exposed overlaying water column community yielded 323 Gbp, of which 80.07% assembled into 3.6-Gbp contigs, with 3.1-Gbp contigs of >1 Kbp. A total of 883 genomes were binned, with only 114 remaining after dereplication. Of these genomes, 62 belonged to families shared with the sediment community, while 52 were water specific. Genomes recovered from the water column belonged to a significantly lower number of phyla (*n* = 27), classes (*n* = 37), orders (*n* = 52), and families (*n* = 79) than those from the euxinic sediments (Table S1). The community exhibited a much lower level of novelty and rarity at the phylum, class, order, and family levels than those of the sediment community (Fig. 2). Water-specific genomes (*n* = 52) belonged mostly to well-characterized microbial lineages, e.g., families *Rhodobacteraceae* and *Rhodospirillaceae* in *Alphaproteobacteria*; families *Thiomicrospiraceae*, *Halothiobacillaceae*, *Acidithiobacillaceae*, *Burkholderiaceae*, *Chromatiaceae*, and *Methylothermaceae* in *Gammaproteobacteria*; families *Sulfurimonadaceae* and *Sulfurovaceae* in phylum *Campylobacterota*; and well described families in the phyla *Bacteroidota* and *Desulfobacterota* (Text S1; Fig. 1; Table S1). Collectively, this information demonstrates a pattern where the intrusion of oxygen is negatively correlated with the presence of previously undescribed and LRD lineages, which are prevalent in the sediment.

### Reductive sulfur processes dominate Zodletone spring sediment communities.

**FIG 4:**
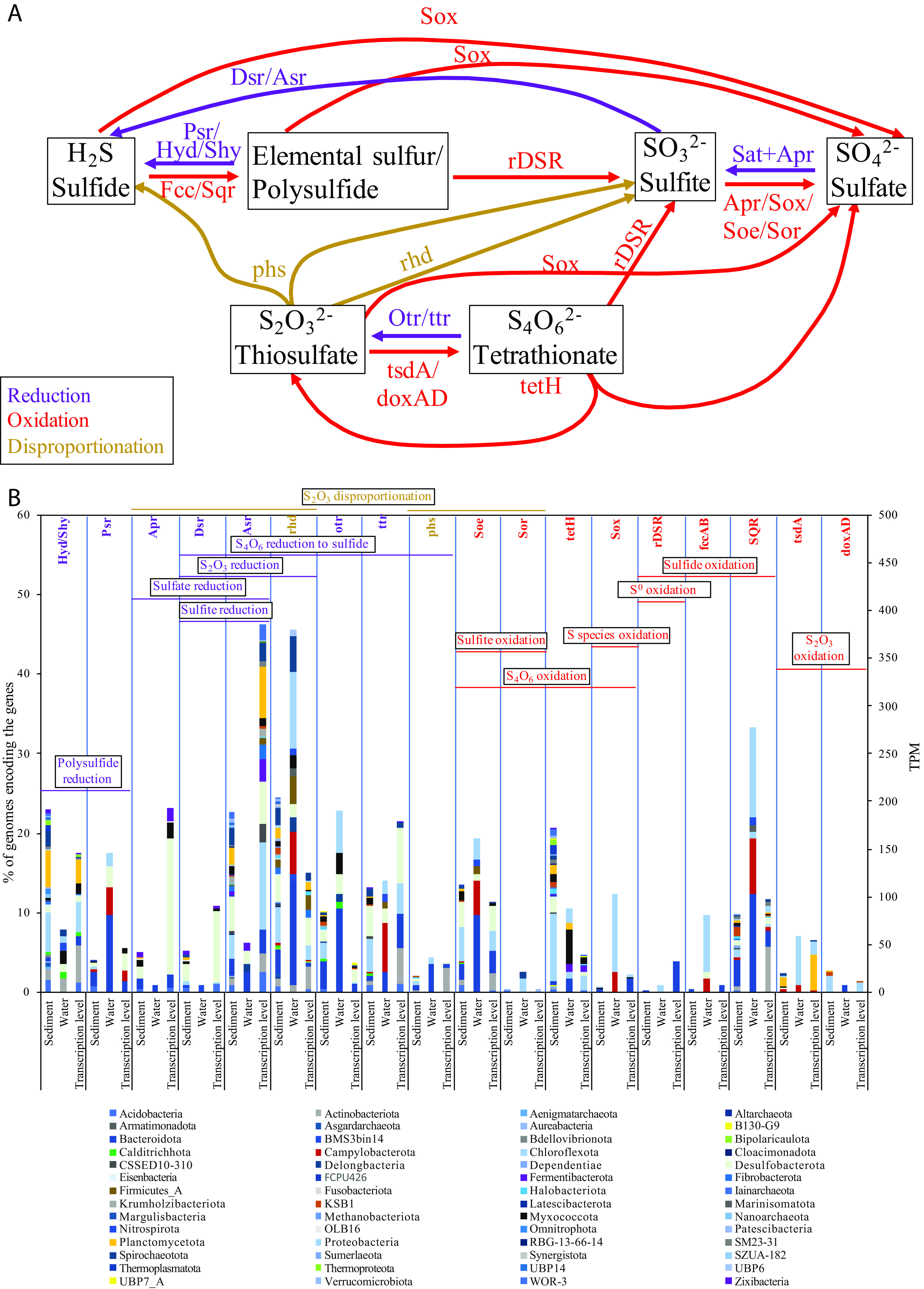
Sulfur cycle in Zodletone spring. (A) Diagram of sulfur transformations predicted to take place in the spring. Different sulfur species are shown in black boxes. Reduction reactions are depicted by purple arrows, oxidation reactions are depicted by red arrows, while disproportionation reactions are depicted by golden-brown arrows. The gene names are shown on the arrows. (B) Phylum-level distribution of the S cycling genes shown at the top of the figure in sediment and water genomes, as well as the transcriptomic data set. Processes involving more than one gene are highlighted by horizontal bars and are color coded by reduction (purple), oxidation (red), or disproportionation (golden brown), with the name of the process shown on top of the horizontal bar. RNA-seq reads were pseudoaligned to the S cycling genes predicted in Zodletone genomes to detect exact matches using Kallisto (95). The transcripts per million are shown on the secondary *y* axis for the gene/group of genes depicted at the top of the figure.

A total of 149 genomes (28.9% of all genomes), belonging to 32 phyla, 51 classes, 69 orders, and 97 families were involved in at least 1 reductive sulfur processes (Fig. 4; see Fig. S4 and Table S2 in the supplemental material). By comparison, only 21 sediment genomes (4.06% of all genomes) encoded at least 1 sulfur oxidation pathway (Fig. 4, Fig. S4; Table S2). The reductive sulfur community in the spring exhibited two unique traits as follows: first, a majority of genomes encoding such capacities belonged to novel (47 genomes) or LRD (66 genomes) lineages (Fig. 4, Fig. S4), and second, sulfite, polysulfide, thiosulfate, and tetrathionate reduction capacities appear to be more prevalent than sulfate-reduction capacities in the sediment genomes.

10.1128/mbio.00016-22.5 FIG S4 Family-level distribution of the genomes involved in S cycling in the spring. The maximum likelihood tree was constructed in FastTree ^40^ based on the concatenated alignments of 120 single-copy genes obtained from GTDB-TK ^41^. The branches represent family-level taxonomy and are color coded by phylum. For phyla with 2 families or less involved in S cycling, branches are labeled as Phylum_Class_Order_Family. For phyla where 3 or more families are involved in S cycling, the phylum is shown at the base of the colored wedge and the branches are labeled as Class_Order_Family. Lineages staring with ZN depict novel lineages as follows: ZNC, novel class; ZNO, novel order; and ZNF, novel family. Bootstrap support values are shown as bubbles for nodes with >70% support. Tracks around the tree represent (from innermost to outermost) the following: heatmap for the number of genomes in each family, abundance in GTDB based on the number of available genomes (abundant with more than 5 genomes, rare with 5 genomes or less, and novel with no genomes in GTDB), pie charts of the breakdown of the number of genomes in the sediment (cyan) versus water (magenta), sulfur reduction pathways (5 tracks in purple), thiosulfate disproportionation pathways (1 track in golden brown), sulfur oxidation pathways (7 tracks in red), and substrates predicted to support growth depicted by colored stars (cyan, sugars; lime green, complex carbohydrates; magenta, amino acids; orange, proteins; purple, CO_2_ fixation; red, short-chain fatty acids (SCFA); blue, beta oxidation of long-chain fatty acids; brown, anaerobic benzoate/aromatic hydrocarbon degradation; black, anaerobic alkane degradation; and gray, hydrogen oxidation). Download FIG S4, PDF file, 1.8 MB. Copyright © 2022 Hahn et al. 2022 Hahn et al. https://creativecommons.org/licenses/by/4.0/ This content is distributed under the terms of the Creative Commons Attribution 4.0 International license.

10.1128/mbio.00016-22.9 TABLE S2 S-cycling genes predicted in Zodletone genomes. Genes are shown in the table header, and actual gene names are shown in the corresponding cells. Download Table S2, XLSX file, 0.1 MB. Copyright © 2022 Hahn et al. 2022 Hahn et al. https://creativecommons.org/licenses/by/4.0/ This content is distributed under the terms of the Creative Commons Attribution 4.0 International license.

Sulfate reduction capacity was encoded in only 18 sediment genomes (Fig. 4, Fig. S4) but exhibited a unique community composition, when compared with well-studied marine and terrestrial habitats (1, 2, 4, 26). Sulfate reduction capacities were observed in mostly previously undescribed or LRD lineages within the *Zixibacteria*, *Acidobacteriota* (members of family UBA6911, equivalent to *Acidobacteria* group 18), *Myxococcota*, *Bacteroidota*, *Planctomycetota*, and candidate phylum OLB16 (1 genome), as well as rare and novel lineages within *Desulfobacterota* (Fig. 4, Fig. S4; see Fig. S5a in the supplemental material; Table S2).

10.1128/mbio.00016-22.6 FIG S5 Expanding lineages mediating reductive sulfur-cycling processes in the tree of life. Annotree ^42^ was queried at the family level for the presence of sulfate reduction genes (combined search for the genes AprA, AprB, Sat, QmoA, QmoB, QmoC, DsrA, DsrB, and DsrC) (A), sulfite reduction genes using both the DSR system (combined search for the genes DsrA, DsrB, and DsrC (B), excluding all duplicate hits from A and excluding all hits from phyla known to use the system in the oxidative direction (*Proteobacteria*, *Nitrospira*, and *Chlorobiaceae*), and the ASR system (combined search for the genes AsrA, AsrB, AsrC, HdrA, HdrB, and HdrC); polysulfide reduction genes (combined search for the genes PsrA, PsrB, and PsrC) (C), thiosulfate reduction (combined search for the genes PhsA, PhsB, and PhsC, combined with either DsrA, DsrB, and DsrC or AsrA, AsrB, and AsrC) and thiosulfate disproportionation (combined search for the genes PhsA, PhsB, and PhsC, combined with either AprA, AprB, and Sat or SoeA, SoeB, and SoeC, or SorA) (D), and tetrathionate reduction (combined search for the genes TtrA, TtrB, and TtrC) (E). For each of these searches, all hits were downloaded, sorted, and parsed to keep one representative from each family with a hit. The GTDB accession IDs for all such representatives were used to extract their 120 single-copy protein concatenated alignment available from the GTDB downloads repository (https://data.gtdb.ecogenomic.org/releases/release95/95.0/genomic_files_reps/). They were then combined with the concatenated alignments for family-level representatives in Zodletone with genomic evidence of the corresponding sulfur reductive. The maximum likelihood trees were constructed in FastTree ^40^. The branches represent family-level taxonomy and are color coded by phylum. Zodletone lineages staring with ZN depict novel lineages as follows: ZNC, novel class; ZNO, novel order; and ZNF, novel family. The track around the tree represents the distribution of each family-level representative as follows: only encountered in Annotree, black; only encountered in Zodletone spring sediment, blue; and encountered in both, red. By comparing the number of lineages with the blue track (only encountered in Zodletone) to the combined number of lineages with the black and red tracks, the level of expansion of lineages mediating reductive sulfur-cycling processes in the tree of life can be deduced as follows: with regard to sulfate reduction (A), and as stated in the results section of the main text, lineages encountered in Zodletone spring sediment encoded minimal sulfate reduction capacities. Only 7 new lineages were added to an existing list of 90 lineages in the bacteria tree of life (increase by 7.7%). On the other hand, with regard to sulfite reduction (B), representatives of 71 new families were added to a list of 76 families already known to carry this function (increase by 93.4%). For polysulfide reduction (C), thiosulfate reduction/disproportionation (D), and tetrathionate reduction (D), increases of 11.8% (12 new lineages added to 102 already known), 30.4% (7 new lineages added to 23 already known), and 34.7% (43 new lineages added to 124 already known), respectively, were encountered. Download FIG S5, PDF file, 2.3 MB. Copyright © 2022 Hahn et al. 2022 Hahn et al. https://creativecommons.org/licenses/by/4.0/ This content is distributed under the terms of the Creative Commons Attribution 4.0 International license.

Sulfite (but not sulfate) reduction via the DsrAB+DsrC+DsrKMJOP system was identified in only 8 genomes belonging to 7 families within the phyla *Planctomycetes*, *Chloroflexota*, *Spirochaetota*, and *Desulfobacterota* (Fig. 4, Fig. S4 and S5b; see Fig. S6a in the supplemental material; Table S2). On the other hand, the sulfite reduction capacity within Zodletone spring sediment solely via the Asr/Hdr system was rampant, being encountered in 104 genomes belonging to 28 phyla, 43 (8 novel and 9 LRD) classes, 56 (18 novel and 12 LRD) orders, and 72 (31 novel and 25 LRD) families (Fig. 4, Fig. S4 and S6b; Table S2), with a gene organization of the *asr* locus adjacent to the *hdr* locus in the majority of genomes (Fig. S6b). Asr-encoding genomes in the sediment included members of previously undescribed and LRD lineages within the *Chloroflexota*, *Desulfobacterota*, *Planctomycetota*, and *Bacteroidota*. The capacity was also rampant in the yet-uncultured bacterial phyla, of which many have a fairly limited global distribution (e.g., the candidate phyla CSSED10-310, FCPU426, RBG-13-66-14, SM23-31, SZUA-182, UBP14, *Aureabacteria*, *Sumerlaeota*, and *Krumholzibacteriota*). Zodletone dissimilatory sulfite reductase (Fig. S6a) and the anaerobic sulfite reductase (Fig. S6b) sequences clustered with reference sequences from the same phylum, generally showing no evidence of LGT.

10.1128/mbio.00016-22.7 FIG S6 Phylogenetic affiliation and contig organization of selected sulfur reduction proteins. Phylogeny of the dissimilatory sulfite reductase DsrAB (EC 1.8.99.5) concatenated proteins (A), anaerobic sulfite reductase subunit B AsrB (B), polysulfide reductase subunit gamma PsrC (C), thiosulfate reductase cytochrome b subunit PhsC (D), and octaheme tetrathionate reductase Otr (E). Alignments were created in MAFFT ^43^ and maximum likelihood trees were constructed in RaxML ^44^. Bootstrap support values are shown as bubbles for nodes with >50% support. Branches and branch labels are color coded by phylum for Zodletone sequences. Branch labels depict classification to family level followed by the NCBI genome accession number. Reference sequences are shown in black with the Uniprot accession numbers. Contig organizations of the DSR and ASR loci in selected Zodletone genomes are shown to the right of the trees in A and B. Genes are color coded as shown in the top right corner. Unrelated genes are shown by gray arrows. Gene maps were created in R using the package genoplotR ^45^. Phylum/class classification is depicted to the right of the trees in C to E. Download FIG S6, PDF file, 2 MB. Copyright © 2022 Hahn et al. 2022 Hahn et al. https://creativecommons.org/licenses/by/4.0/ This content is distributed under the terms of the Creative Commons Attribution 4.0 International license.

Sulfur (polysulfide) reduction capacities were observed in 20 Zodletone sediment genomes that encoded *psrABC* genes (Fig. 4, Fig. S4, S5c, and S6c; Table S2). In addition, representatives of the cytoplasmic sulfurhydrogenase I (HydABCD system) and/or II (ShyABCD system) were identified in 119 Zodletone sediment genomes (Fig. 4). However, the direct involvement of these enzymes in an ETS-associated respiration is not yet clear (Text S1).

Sediment genomes also encoded thiosulfate disproportionation and reduction capacities. The quinone-dependent membrane-bound molybdopterin-containing thiosulfate reductase PhsABC was encoded in 11 genomes belonging to 6 phyla (Table S2; Fig. S6d). Within these genomes, only two (a *Chloroflexota* family UBA6092 genome and a *Desulfatiglandales* family HGW15 genome) also encoded a dissimilatory sulfite reductase (the Asr system) akin to the *Gammaproteobacteria* thiosulfate-disproportionating pure culture members (27), where the final products of thiosulfate disproportionation are expected to be only hydrogen sulfide (Fig. 4, Fig. S4 and S5d; Table S2). On the other hand, 5 of the 11 phsABC-encoding Zodletone genomes also encoded the sulfite dehydrogenase SoeABC system, akin to *Desulfobacterota* and *Firmicutes* pure culture members, where the final products of thiosulfate disproportionation are expected to be both hydrogen sulfide and sulfate (28, 29) (Fig. 4, Fig. S4 and S5d; Table S2).

In addition to the phsABC system, 14 Zodletone genomes belonging to 6 phyla encoded a rhodanase-like enzyme (EC 2.8.1.1 or EC 2.8.1.3) for thiosulfate disproportionation, as well as enzymes for both sulfite oxidation (by means of reversal of sulfate reduction via Sat+AprAB or the sulfite dehydrogenase SoeABC), and sulfite reduction (via the dissimilatory sulfite reductases Dsr or Asr), where the final products of thiosulfate disproportionation are expected to be both hydrogen sulfide and sulfate (30–34) (Fig. 4, Fig. S4 and S5d; Table S2).

Tetrathionate reduction capacities were identified in 105 sediment genomes. Seventy-three Zodletone sediment genomes from 14 phyla encoded the octaheme tetrathionate reductase (OTR) enzyme (Table S2; Fig. 4, Fig. S4 and S6e). In addition to Otr, 68 Zodletone genomes from 14 phyla encoded the Ttr enzyme system (Table S2; Fig. 4, Fig. S4 and S5e). As shown previously in Salmonella enterica serovar Typhimurium (27), in the presence of means for thiosulfate disproportionation/reduction and sulfite reduction, the thiosulfate produced as a result of tetrathionate reduction could be further reduced to sulfide. Out of the 105 sediment genomes encoding the Otr, and/or Ttr enzymes, only 12 genomes also encoded thiosulfate and sulfite reduction enzymes.

Within lineages mediating reductive sulfur processes in Zodletone sediments (*n* = 98), a wide range of substrates supporting sulfidogenesis were identified (Table S3; Fig. S4). They included hexoses (26% to 87% of sulfidogenic lineages); pentoses (30% to 41% of sulfidogenic lineages); amino acids and peptides (39% of lineages); short-chain fatty acids, e.g., lactate, propionate, butyrate, and acetate (22% to 73% of lineages); long-chain fatty acids (29% of lineages); aromatic hydrocarbons (3% of lineages); and short-chain alkanes (6% of lineages). Autotrophic capacities with hydrogen as the electron donor were identified in 28% of sulfidogenic lineages.

10.1128/mbio.00016-22.10 TABLE S3 Substrates potentially supporting growth, predicted fermentation end products, and energy conservation pathways predicted from genomic analysis. Download Table S3, XLSX file, 0.1 MB. Copyright © 2022 Hahn et al. 2022 Hahn et al. https://creativecommons.org/licenses/by/4.0/ This content is distributed under the terms of the Creative Commons Attribution 4.0 International license.

### Transcriptomic analysis.

Transcriptional expression of genes involved in S species reduction/disproportionation was analyzed in the spring sediments (Fig. 4). All S species reduction/disproportionation genes discussed above were identified and mapped to 51 distinct phyla. Total transcription levels of the Asr system were 4 times higher than those of the Dsr system, which is consistent with the higher number of Zodletone sediment genomes encoding the Asr system than that of the Dsr system. Asr system genes were mapped to 11 phyla; while DSR genes mapped to 4 phyla (Fig. 4). Sulfate reduction genes (Sat, AprAB, and QmoABC) were also transcribed with major contributions from 4 phyla. Transcription of the thiosulfate disproportionating rhodanese-like (EC 2.8.1.1 or EC 2.8.1.3), thiosulfate reductase *phsABC*, tetrathionate reduction genes *ttrABC*, octaheme tetrathionate reductase *otr*, *psrABC* for polysulfide reduction, and cytoplasmic sulfurhydrogenases I and II (*hyd/shy* systems) were also identified (Fig. 4; Text S1, for detailed contributions of taxa).

### Oxidative sulfur processes dominate the Zodletone water community.

Reductive sulfur processes were extremely sparse in the water community (Fig. 4, Fig. S4; Table S2; Text S1). In contrast, oxidative sulfur processes dominated the water community, with pathways encoding sulfide, sulfur, thiosulfate, tetrathionate, and/or sulfite oxidation to sulfate present in 59/114 (51.8%) of water genomes, belonging to 13 phyla, 16 classes, 25 orders, and 43 families, respectively. The oxidative sulfur community in the water belonged to mostly well-characterized lineages (Table S2; Fig. 4, Fig. S4), with only 8 and 10 genomes involved in oxidative sulfur processes belonging to previously undescribed and LDR families, respectively. A complete SOX system, putatively mediating oxidation of a wide range of reduced sulfur-species to sulfate, was encoded in genomes belonging to well-characterized families within *Proteobacteria* (11 genomes in *Acidithiobacillaceae*, *Burkholderiaceae*, *Halothiobacillaceae*, *Rhodobacteraceae*, and *Thiomicrospiraceae*) and *Campylobacterota* (3 genomes in the family *Sulfurimonadaceae*) (Table S2; Fig. 4, Fig. S4). The capacity for sulfide oxidation to sulfur (sulfide dehydrogenase and/or the sulfide:quinone oxidoreductase Sqr) was encoded in 39 water genomes (Text S1; Table S2; Fig. 4, Fig. S4). Only 2 of the above 39 genomes (a *Proteobacteria* genome and a *Nitrospirota* genome) encoded the capacity to further oxidize the sulfur/polysulfide to sulfite via the reversal of the Dsr system. The capacity for sulfite oxidation to sulfate via the reversal of AprAB+QmoABC system, the sulfite dehydrogenase (quinone) SoeABC, or the sulfite dehydrogenase (cytochrome) SorAB system was encoded in 1, 22, and 3 genomes, respectively (Text S1; Table S2; Fig. 4, Fig. S4). Finally, for thiosulfate oxidation, eight water genomes (*Proteobacteria* and *Flavobacteriaceae*) encoded thiosulfate to tetrathionate oxidation capacities via either the thiosulfate dehydrogenase *tsdA* (EC 1.8.2.2) or *doxAD* (EC 1.8.5.2). Two of these 8 genomes also encoded tetrathionate hydrolase (*tetH*) (35) that is known to cleave tetrathionate to thiosulfate, sulfur, and sulfate (Text S1; Table S2; Fig. 4, Fig. S4). Simultaneous identification of the SOX system and both forms of sulfide dehydrogenase (fccAB and Sqr) imply that these two genomes encode the capacity for complete thiosulfate oxidation to sulfate.

## DISCUSSION

The microbial community in Zodletone spring sediments exhibited a high level of phylogenetic diversity, novelty, and rarity (Fig. 1 to 3, Fig. S3). Conversely, representatives of lineages that predominate in most present earth environments, e.g., *Proteobacteria*, *Firmicutes*, and *Cyanobacteria*, were absent or extremely sparse within the sediments. The community in the spring sediments was also characterized by a high proportion of SRM and the prevalence of lineages mediating the reduction of sulfur cycle intermediates (sulfite, thiosulfate, tetrathionate, and elemental sulfur) over sulfate reducers. Many of the organisms mediating reductive sulfur-cycling processes belonged to novel and LRD lineages (Fig. S4), hence expanding the range of SRM within the tree of life (Fig. S5).

What drives the assembly, propagation, and maintenance of such a diverse, novel, and distinct community in the spring sediments? The high level of diversity, novelty, and rarity within Zodletone spring sediment SRM community could be attributed to two main factors. First, a wide range of sulfur cycle intermediates are available in concentrations much higher than sulfate, in contrast to sulfate predominance in current ecosystems (2). Such a pattern selects for a more diverse community of SRM in the spring than that of predominantly sulfate-driven marine and freshwater ecosystems (Fig. S4). Second, additional factors usually constraining SRM growth in several habitats, such as diel or seasonal intrusion of oxygen, Fe and NO_3_ (1, 36–38), recalcitrance of available substrates (6, 39, 40), temperature (41, 42), pH (26, 43, 44), salinity (45), and pressure extremes (39, 46), or combinations thereof, are absent in the spring. Therefore, while the reductive global sulfur cycle appears to be dominated by a few sulfate-reducing lineages within *Desulfobacterota*, and to a lesser extent *Firmicutes*, as well as *Thermodesulfobacteria* and *Archaeoglobus* in high-temperature habitats, the SRM community in Zodletone is extremely diverse, encompassing a wide range of previously undescribed and LRD lineages (Fig. 4, Fig. S4, S5, and S6).

Sulfate-reducing organisms are the most prevalent component of the reductive sulfur cycle in most marine and aquatic ecosystems. Aspects of the ecology (2), physiology (47), and biochemistry (48–50) of dissimilatory sulfate reduction have been investigated extensively (51). While not the most prevalent process, the sulfate-reducing community in Zodletone spring sediment exhibited a unique composition, with members of *Zixibacteria*, *Acidobacteriota*, *Myxococcota*, *Bacteroidota*, *Planctomycetota*, and candidate phylum OLB16 constituting the major players, as well as rare and novel lineages within *Desulfobacterota* (*Desulfatiglandales* and order C00003060), with scarce representation of canonical *Desulfobacterota* sulfate reducers (1 genome). While the identification of the dissimilatory sulfate-reducing machinery in some of these lineages (e.g., *Zixibacteria*, *Acidobacteriota*, and *Planctomycetota*) has been shown before (52–54), these members rarely appear to be the dominant players in a single ecosystem.

Compared with sulfate reduction, the ecology and diversity of microbial dissimilatory sulfite reduction has not been studied extensively. The biochemistry of the process has been examined in sulfate reducers, when grown on sulfite (51), as well as in a few other dedicated sulfite reducers, such as members of *Desulfitobacterium* (55), Salmonella (56), *Shewanella* (57), and *Wolinella* (58). A recent study suggested the importance and the ancient nature of sulfite reduction in an extreme thermophilic environment in a limited diversity biofilm (59). We document a plethora of microorganisms within the phyla *Planctomycetes*, *Chloroflexota*, *Spirochaetota*, and *Desulfobacterota* encoding the dissimilatory sulfite reductase DSR, as well as 72 additional families (31 novel and 25 LRD) encoding the anaerobic sulfite reductase. These organisms expand the known sulfite reduction capacity within the domain *Bacteria* (Fig. S5). Furthermore, the novelty or rarity of some of these families is a reflection of the dearth of current habitats that could support this mode of metabolism, once predominant on ancient earth.

The bulk of knowledge on thiosulfate reduction and or disproportionation comes from studies in pure cultures, e.g., members of *Desulfobulbaceae* (e.g., *Desulfocapsa*) (28, 60) and the genera *Desulfovibrio* and *Desulfomonile* (61–63) within *Desulfobacterota*, the gammaproteobacterium Pantoea agglomerans (64), and members of *Thermodesulfobacteria* (65) and *Firmicutes* (29). Radioisotope tracing of different sulfur atoms showed a significant contribution of thiosulfate disproportionation to the sulfur cycle in marine (66), as well as freshwater, sediments (67). However, the lack of a marker gene for the process hinders ecological culture-independent studies. Similar to sulfite, the high levels and constant generation of thiosulfate in Zodletone sediments sustain a highly diverse thiosulfate-reducing (thiosulfate reductase plus a sulfite reduction complex) or thiosulfate-disproportionating (thiosulfate reductase plus both sulfite reduction and sulfite oxidation systems) community with major contributions from novel or rare families in *Acidobacteriota*, *Chloroflexota*, *Desulfobacterota*, KSB1, *Myxococcota*, and *Spirochaetota*. Finally, the extremely high levels of zero valent sulfur, available as soluble polysulfide, result in enriching the community with a plethora of polysulfide-reducing organisms.

As described above, this study infers that the microbial communities thriving under ancient conditions of anoxia and a high proportion of sulfur cycle intermediates were extremely diverse. In comparison, communities in the oxygen-exposed water column were markedly less diverse. What drives this drastic shift in diversity and community structure? We argue that oxygen introduction into the system is responsible for such a shift, as evident by the shift to oxidative sulfur processes in the water samples. The prevailing conditions in the water column are hence more akin to microbial communities that would thrive in sulfur-rich yet air-exposed habitat on the current earth.

The comparison presented here between both communities could demonstrate putatively how ancient metabolic pathways and lineages mediating them have been curtailed due to oxygen evolution and predominance in the current surficial earth. The evolution of oxygenic photosynthesis has led to the steady and inexorable accumulation of O_2_ in Earth’s atmosphere (the great oxidation event [GOE]), with the rise of atmospheric O_2_ to 1% to 5% of current levels between 2.4 and 2.1 billion years (Gyr) ago, and its accumulation to values comparable to modern values 500 to 600 million years ago (Mya) (68). Due to the expected sensitivity and lack of adaptive mechanisms to cope with atmospheric oxygen in multiple strict anaerobes, as well as the chemical instability of multiple S species in an oxygenated atmosphere, the GOE exerted a profound negative impact on anaerobic surficial life forms (the oxygen catastrophe) leading to the first and arguably most profound extinction event in earth’s history (68). In addition to suppressing anaerobiosis in atmospherically exposed habitats, the GOE also led to a significant change in the S cycle, from one based on atmospheric inputs to one dependent on oxidative weathering leading to the release of a huge amount of sulfate derived from the oxidation of pyrite and the dissolution of sulfate minerals (69), hitherto a minor by-product of Archean abiotic and biotic reactions (15, 70). Therefore, it appears that the loss of niches associated with geological transformations could be one of the possible explanations for high extinction rates for microorganisms on earth, as well as the constant identification of rare, novel taxa within anaerobic settings. It is notable that phylogenetically novel branches with extremely rare distribution on earth (defined as phyla with 5 genomes or less in GTDB) have been identified consistently in anaerobic habitats.

In summary, by examining microbial diversity in Zodletone spring, we greatly expand the overall diversity within the tree of life via the discovery and characterization of a wide range of novel lineages and significantly enrich the representation of a wide range of LRD lineages. We also describe a unique sulfur-cycling community in the spring that is largely dependent on sulfite, thiosulfate, sulfur, and tetrathionate, rather than sulfate, as an electron acceptor. Given the remarkable similarity to conditions prevailing prior to the GOE, we consider the spring an invaluable portal with which to investigate the community thriving on the earth’s surface during these eras and posit that GOE precipitated the near extinction of a wide range of phylogenetically distinct oxygen-sensitive lineages and drastically altered the reductive sulfur-cycling community from sulfite, sulfur, and thiosulfate reducers to predominantly sulfate reduction in the current earth.

## MATERIALS AND METHODS

### Site description and geochemistry.

Zodletone spring is located in the Anadarko Basin of western Oklahoma (N34.99562° W98.68895°). The spring arises from underground, where water is pumped out slowly along with sediments. Sediments settled at the source of the spring, a boxed square of 1 m^2^ (Fig. S1), are overlaid with water that collects and settles in a concrete pool erected in the early 1900s. The settled water is 50 cm deep above the sediments and is exposed to atmospheric air. Water and sediments originating from the spring source are highly reduced due to the high dissolved sulfide levels (8 to 10 mM) in the spring sediments. Microsensor measurements show a completely anoxic (oxygen levels of <0.1 μM) and highly reduced source sediments. Oxygen levels slowly increase in the overlaid water column from 2 to 4 μM in the 2 mm above the source to complete oxygen exposure at the top of the water column (19). The spring geochemistry has been monitored regularly during the last 2 decades (19, 20, 71) and is remarkably stable. The spring is characterized by low levels of sulfate (50 to 94 μM), with higher levels of sulfite (0.21 mM), elemental sulfur (0.1 mM), and thiosulfate (0.52 mM) (21, 71).

### Sampling.

Samples were collected from the source sediments and standing overlaid water in sterile containers and kept on ice until they were transported to the lab (∼2-h drive), where they were processed immediately. For metatranscriptomics, samples were collected at three different time points, namely, morning (9:15 a.m.), afternoon (2:30 p.m.), and evening (5:30 p.m.) in June 2019. They were stored on dry ice and then transferred to the lab where they were stored at −80°C until being processed for RNA extraction within a week.

### Nucleic acid extraction.

DNA was extracted directly from 0.5 g of source sediments. For water samples, water was filtered on 0.2-μm sterile filters. DNA was directly extracted from filters (20 filters, 10 L of water samples). Extraction was conducted using the DNeasy PowerSoil kit (Qiagen, Valencia, CA, USA). RNA was extracted from 0.5-g sediment samples using RNeasy PowerSoil total RNA kit (Qiagen) according to the manufacturer;s instructions.

### 16S rRNA gene amplification, sequencing, and analysis.

Triplicate DNA extractions were performed for both sediment and water samples from the Zodletone spring. To characterize the microbial diversity based on 16S rRNA gene sequences, we used the Quick-16S next-generation sequencing (NGS) library prep kit (Zymo Research, Irvine CA), following the manufacturer’s protocol. For amplification of the V4 hypervariable region, we used a mix of modified versions of primers 515F-806R (72), tailored to provide better coverage for several underrepresented microbial lineages. They included 515FY (5′-GTGYCAGCMGCCGCGGTAA) (73), 515F-Cren (5′-GTGKCAGCMGCCGCGGTAA, for *Crenarchaeota*) (74), 515F-Nano (5′-GTGGCAGYCGCCRCGGKAA, for *Nanoarchaeota*) (74), and 515F-TM7 (5′-GTGCCAGCMGCCGCGGTCA for TM7/*Saccharibacteria*) (75) as forward mix and 805RB (5′-GGACTACNVGGGTWTCTAAT) (76) and 805R-Nano (5′-GGAMTACHGGGGTCTCTAAT, for Nanoarchaeota) (74) as reverse mix. Purified barcoded amplicon libraries were sequenced on a MiSeq instrument (Illumina Inc., San Diego, CA) using a v2 500-cycle kit, according to the manufacturer’s protocol. Demultiplexed forward and reverse reads were imported as paired fastq files into QIIME2 v. 2020.8 (77) for analysis. The DADA2 plugin was used to trim, denoise, pair, purge chimeras, and select amplicon sequence variants (ASVs), using the command “qiime dada2 denoise-paired.” Between 44,000 and 194,000 nonchimeric sequences were obtained for the individual samples. The ASVs were classified taxonomically in QIIME2 using a trained classifier built based on the Silva-138-99 rRNA sequence database. The ASVs were assigned to 1,643 taxonomic categories corresponding to taxonomic level 7 (species and above) and to 932 genera (level 6). Alpha rarefaction curves indicated a saturation of observed sequence features (ASVs) at a sequencing depth of 70,000 to 80,000 sequences.

### Metagenome sequencing, assembly, and binning.

Metagenomic sequencing was conducted using the services of a commercial provider (Novogene, Beijing, China) using two lanes of the Illumina HiSeq 2500 system for each of the water and sediment samples. Transcriptomic sequencing using Illumina HiSeq 2500 2 × 150-bp paired-end technology was conducted using the services of a commercial provider (Novogene Corporation). Metagenomic reads were assessed for quality using FastQC followed by quality filtering and trimming using Trimmomatic v.0.38 (78). High-quality reads were assembled into contigs using MegaHit (v.1.1.3) with minimum kmer of 27, maximum kmer of 127, kmer step of 10, and minimum contig length of 1,000 bp. Bowtie2 was used to calculate sequencing coverage of each contig by mapping the raw reads back to the contigs. Assembled contigs were searched for ribosomal protein S3 (rpS3) sequences using a custom hidden Markov model (HMM) built from Uniprot reference sequences assigned to the KEGG orthologies (Kos) K02982 and K02984 (corresponding to the bacterial, and archaeal RPS3, respectively) using hmmbuild (HMMER 3.1b2). rpS3 sequences were clustered at 99% identity (ID) using CD-HIT as suggested previously for a putative species cutoff for rpS3 data (79). Taxonomic affiliations of rpS3 groups were identified using Diamond BLAST against the GTDB r95 database (24).

Contigs from the sediment and water assemblies were binned into draft genomes using both Metabat (80) and MaxBin2 (81). DasTool was used to select the highest quality bins from each metagenome assembly (82). CheckM was used for the estimation of genome completeness, strain heterogeneity, and contamination (83). Genomic bins showing contamination levels higher than 10% were further refined based on the taxonomic affiliations of the binned contigs, as well as the GC content, tetranucleotide frequency, and coverage levels using RefineM (84). Low-quality bins (>10% contamination) were cleaned by removal of the identified outlier contigs, and the percentage completeness and contamination were again rechecked using CheckM.

### Genome classification, annotation, and metabolic analysis.

Taxonomic classifications followed the Genome Taxonomy Database (GTDB) release r95 (24) and were carried out using the classify_workflow in GTDB-Tk (v.1.1.0) (85). Phylogenomic analysis utilized the concatenated alignment of a set of 120 single-copy bacterial genes and 122 single-copy archaeal genes (24) generated by the GTDB-Tk. A maximum-likelihood phylogenomic tree was constructed in FastTree using the default parameters (86).

### Annotation and metabolic analysis.

Protein-coding genes were predicted using Prodigal (87). GhostKOALA (88) was used for the functional annotation of every predicted open reading frame in every genomic bin and to assign protein-coding genes to KEGG orthologies (KOs).

### Analysis of sulfur-cycling genes.

To identify taxa mediating key sulfur-transformation processes in the spring sediments, we mapped the distribution of key sulfur-cycling genes in all genomes and deduced capacities in individual genomes by documenting the occurrence of entire pathways (as explained below in detail). This information was confirmed subsequently by phylogenetic analysis and examining contiguous gene organization in processes requiring a multisubunit and/or multigene. Furthermore, expression data were used from three time points to identify the fraction of the community that is metabolically actively involved in the process. An analysis of Sulfur (S) cycling capabilities was conducted on individual genomic bins by building and scanning hidden Markov model (HMM) profiles as explained below. To build the sulfur gene HMM profiles, Uniprot reference sequences for all genes with an assigned KO number were downloaded and aligned using Clustal Omega (89), and the alignment was used to build an HMM profile using hmmbuild (HMMER 3.1b2) (90). For genes not assigned a KO number (e.g., *otr*, *tsdA*, and *tetH*), a representative protein was compared against the KEGG database using BLASTP, and significant hits (those with E values of <e-80) were downloaded and used to build HMM profiles as explained above. The custom-built HMM profiles were then used to scan the analyzed genomes for significant hits using hmmscan (HMMER 3.1b2) (90) with the option -T 100 to limit the results to only those profiles with an alignment score of at least 100. Further confirmation was achieved through phylogenetic assessment and tree building procedures, in which potential candidates identified by hmmscan were aligned to the reference sequences used to build the custom HMM profiles using Clustal Omega (89), followed by maximum likelihood phylogenetic tree construction using FastTree (86). Only candidates clustering with reference sequences were deemed true hits and were assigned to the corresponding KO. Details on the genes examined for evidence of sulfate, sulfite, polysulfide, tetrathionate, and thiosulfate reduction; thiosulfate disproportionation; and various sulfur oxidation capacities are provided in the supplemental material (Text S1).

### Phylogenetic analysis and operon organization of S cycling genes.

The phylogenetic affiliation of the S cycling proteins AsrB, Otr, PhsC, PsrC, and DsrAB was examined by aligning the Zodletone genome predicted protein sequences to Uniprot reference sequences using MAFFT (91). The DsrA and DsrB alignments were concatenated in MEGA X (92). All alignments were used to construct maximum likelihood phylogenetic trees in RAxML (93). The R package genoPlotR (94) was used to produce gene maps for the DSR and ASR loci in Zodletone genomes using the Prodigal predicted gene starts, ends, and strand direction.

### Transcription of sulfur-cycling genes.

A total of 21.4 million, 27.9 million, and 22.5 million 150-bp paired-end reads were obtained from the morning, afternoon, and evening transcriptome sequencing (RNA-seq) libraries. Reads were pseudoaligned to all Prodigal-predicted genes from all genomes using Kallisto with default settings (95). The calculated transcripts per million (TPM) were used to obtain total transcription levels for genes identified from genomic analysis as involved in S cycling in the spring.

### Additional metabolic analysis.

For all other non-sulfur-related functional predictions, combined GhostKOALA outputs of all genomes belonging to a certain order (for orders with 5 genomes or less; *n* = 206) or family (for orders with more than 5 genomes; *n* = 85) were checked for the presence of groups of KOs constituting metabolic pathways (https://github.com/nohayoussef/Zodletone_Metagenomics). The list of these 291 lineages is shown in Table S3. The presence of at least 80% of KOs assigned to a certain pathway in at least one genome belonging to a certain order/family was used as an indication of the presence of that pathway in that order/family. Such criteria were used for the prediction of autotrophic capabilities, as well as catabolic heterotrophic degradation capabilities of sugars, amino acids, long-chain fatty acids, short-chain fatty acids, anaerobic benzoate degradation, anaerobic short-chain alkane degradation, aerobic respiration, nitrate reduction, nitrification, and chlorophyll biosynthesis. Glycolytic and fermentation capabilities were predicted by feeding the GhostKOALA output to KeggDecoder (96). Proteases, peptidases, and protease inhibitors were identified using BLASTP against the MEROPS database (97), while CAZymes (glycoside hydrolases [GHs], polysaccharide lyases [PLs], and carbohydrate esterases [CEs]) were identified by searching all open reading frames (ORFs) from all genomes against the dbCAN hidden Markov model v9 (98) (downloaded from the dbCAN Web server in September 2020) using hmmscan. FeGenie (99) was used to predict the presence of iron reduction and iron oxidation genes in individual bins.

### Data availability.

The whole-genome shotgun project was submitted to GenBank under BioProject identifier (ID) PRJNA690107 and BioSample IDs SAMN17269717 (for the sediment metagenome) and SAMN17269718 (for the water metagenome). The individual assembled MAGs have been deposited at DDBJ/ENA/GenBank under accession numbers JAFFZZ000000000 to JAFGPI000000000. The versions described in this paper are the first versions, JAFFZZ010000000 to JAFGPI010000000. Metagenomic raw reads for the sediment and the water are available under SRA accession numbers SRX9813571 and SRX9813572. RNA-seq reads generated in this study are available under SRA accession numbers SRX9810743, SRX9810744, and SRX9810745 for the morning, afternoon, and evening samples, respectively.
