# Supplementary figures and images for: Microbial Diversity and Sulfur Cycling in an Early Earth Analogue: From Ancient Novelty to Modern Commonality
Source: mBio. 2022 Mar 8;13(2):e00016-22. doi: 10.1128/mbio.00016-22 (PMC9040765; doi:10.1128/mbio.00016-22)

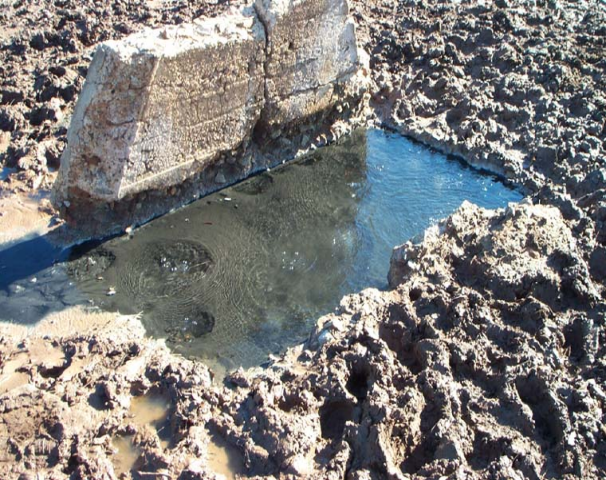

Supplement: FIG S1 [file mbio.00016-22-sf001.pdf]

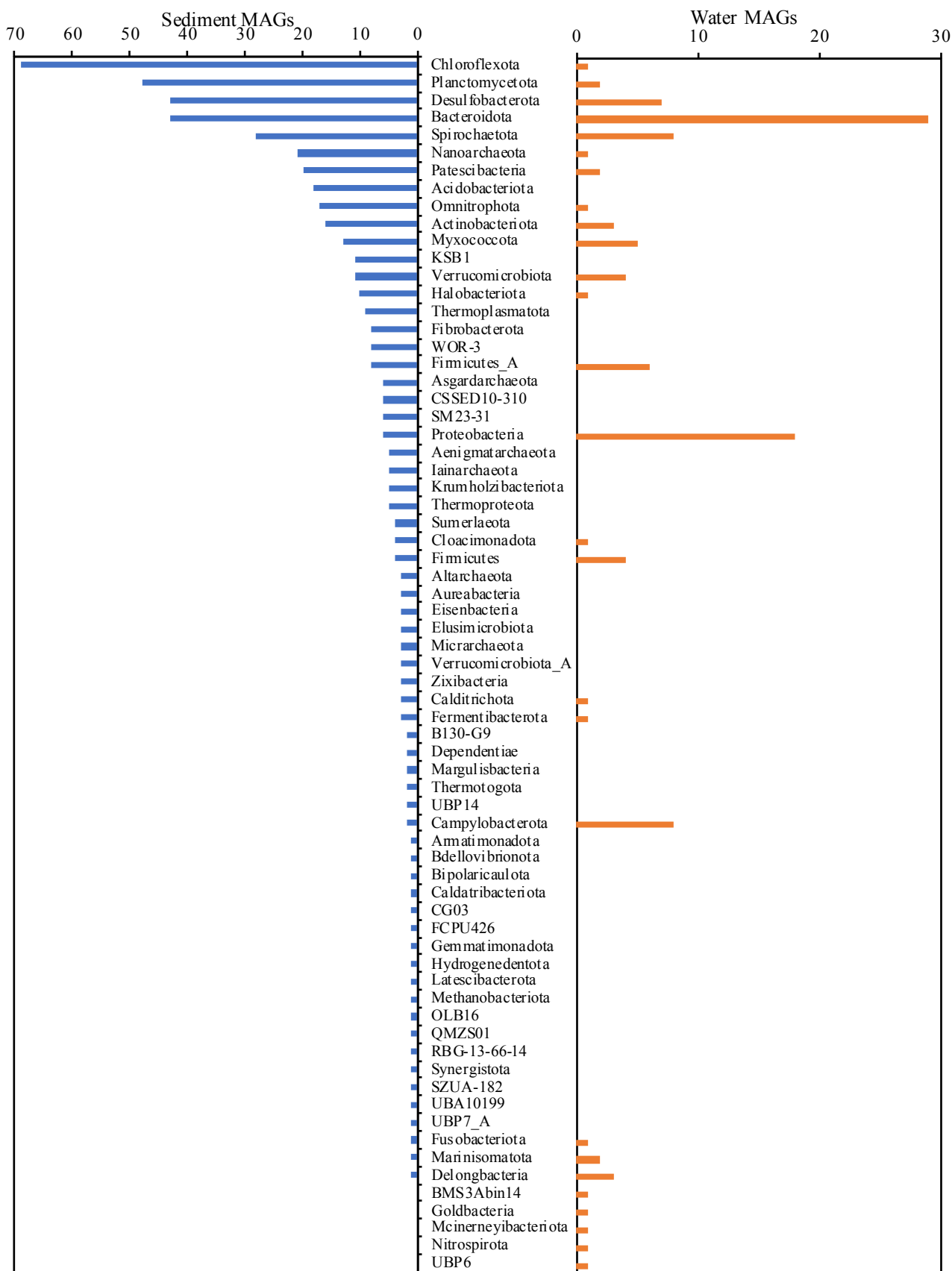

Supplement: FIG S3 [file mbio.00016-22-sf003.pdf]

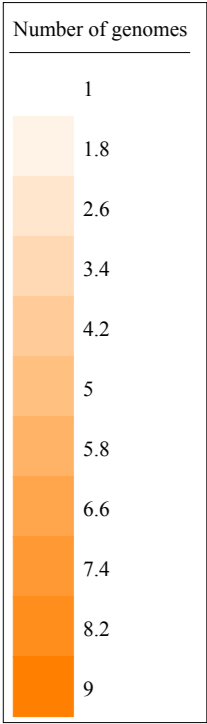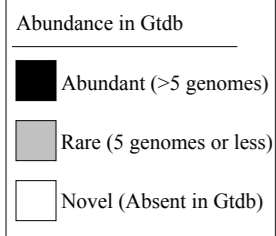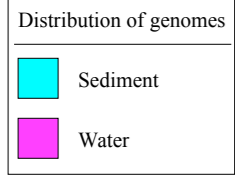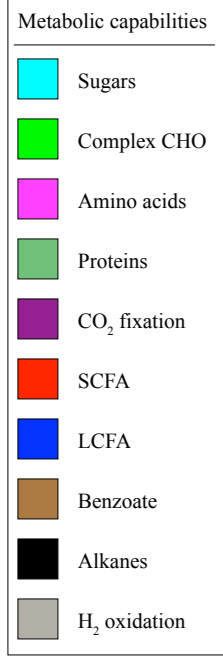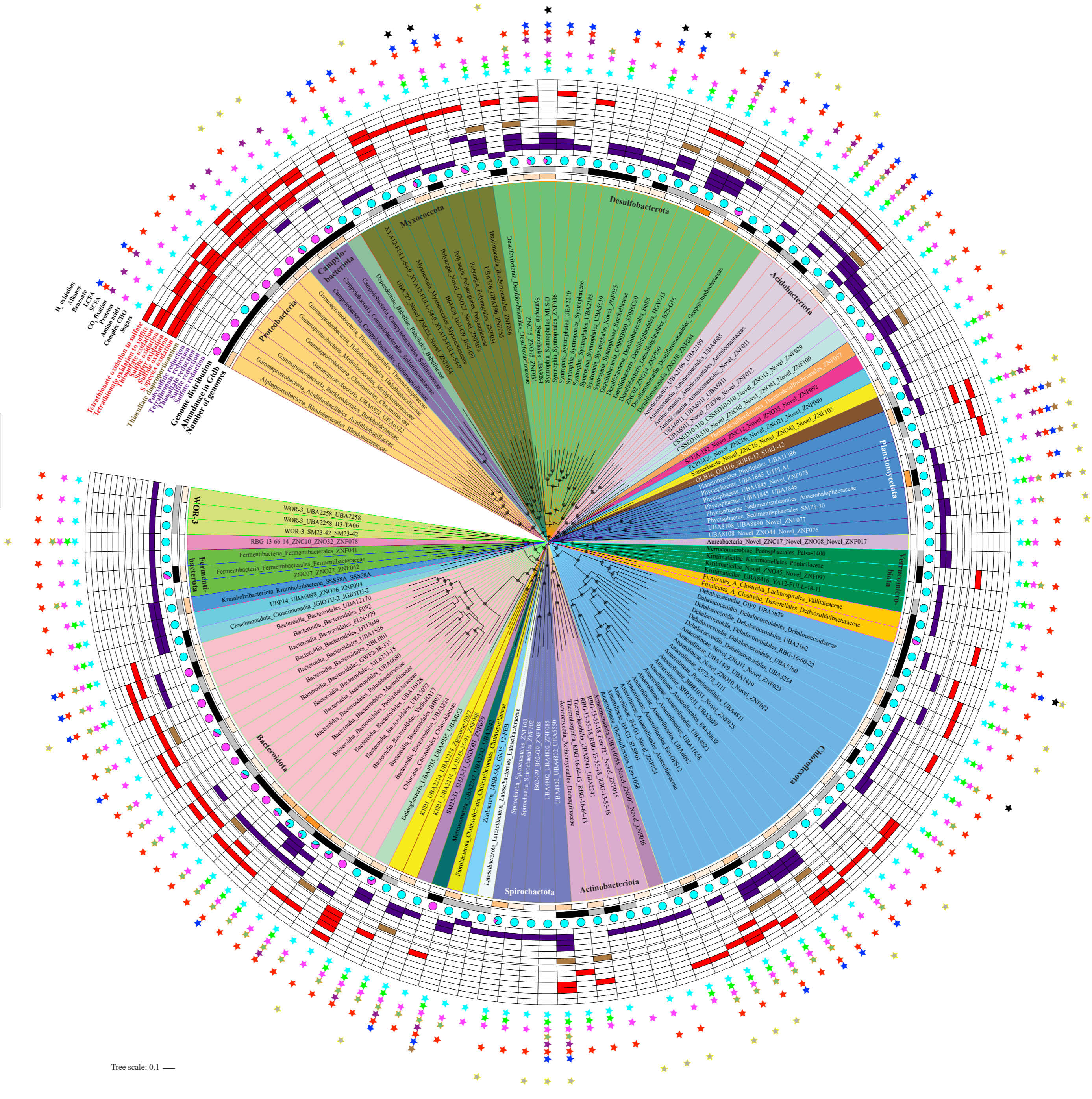

Supplement: FIG S4 [file mbio.00016-22-sf004.pdf]

A

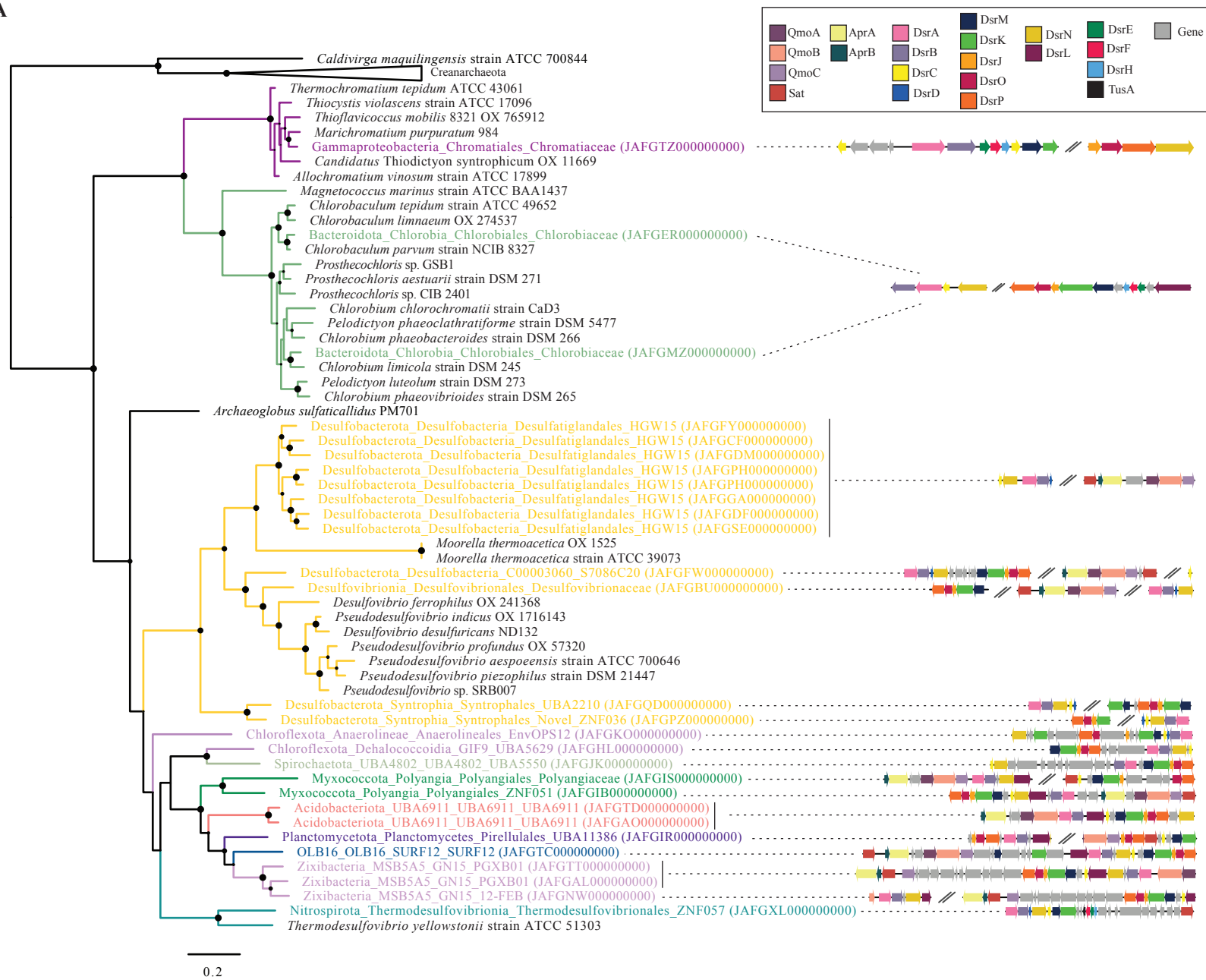

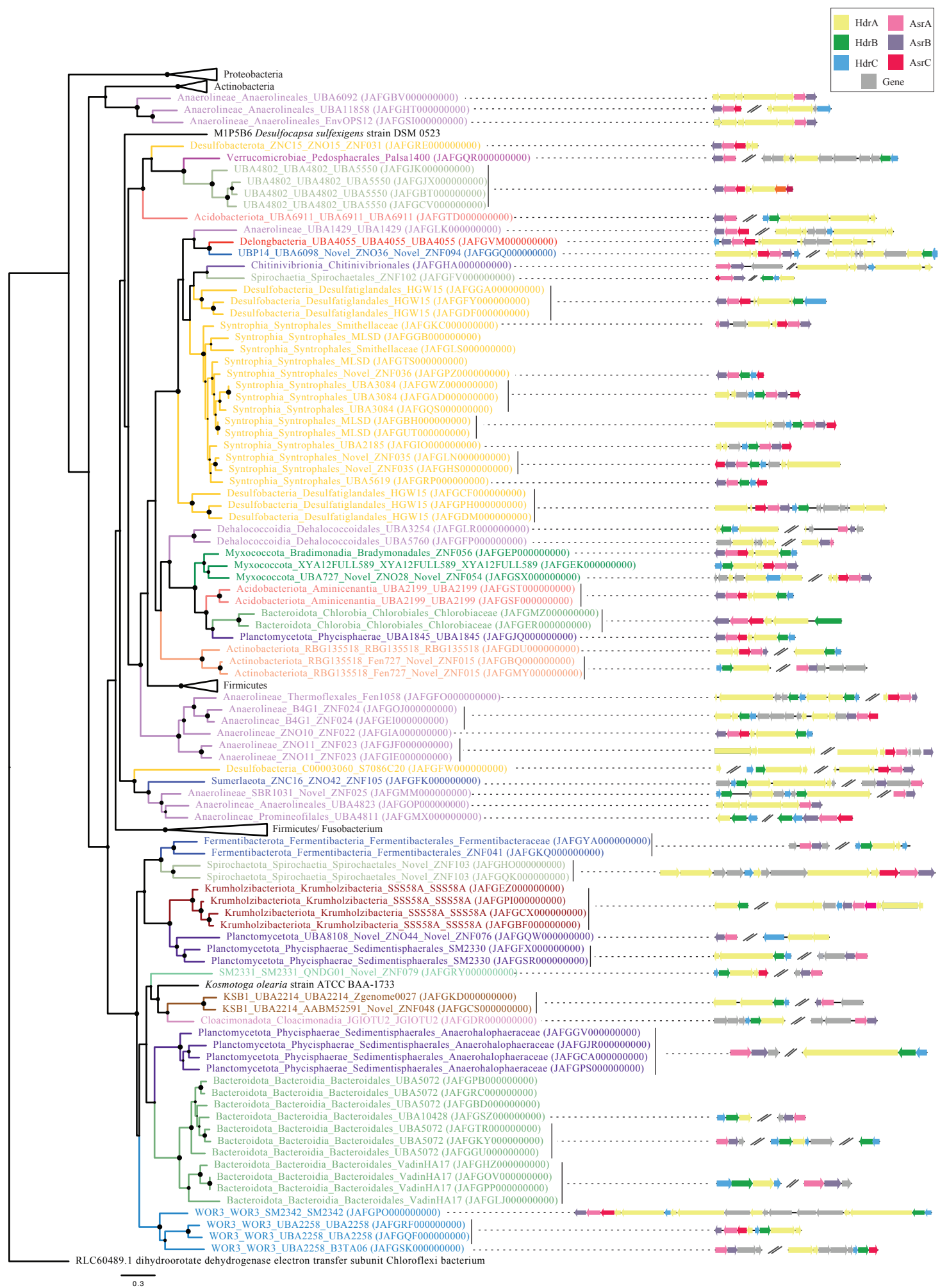

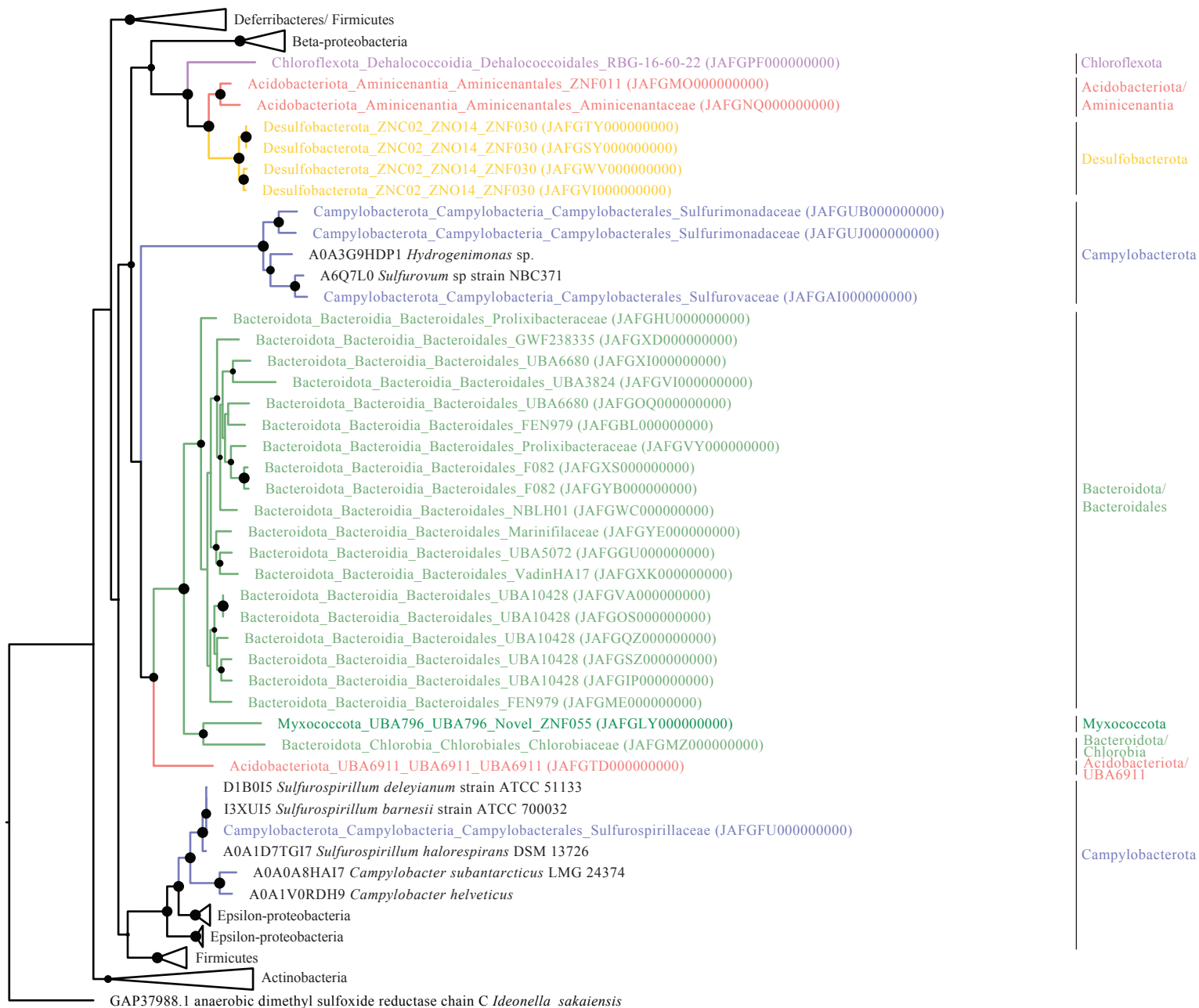

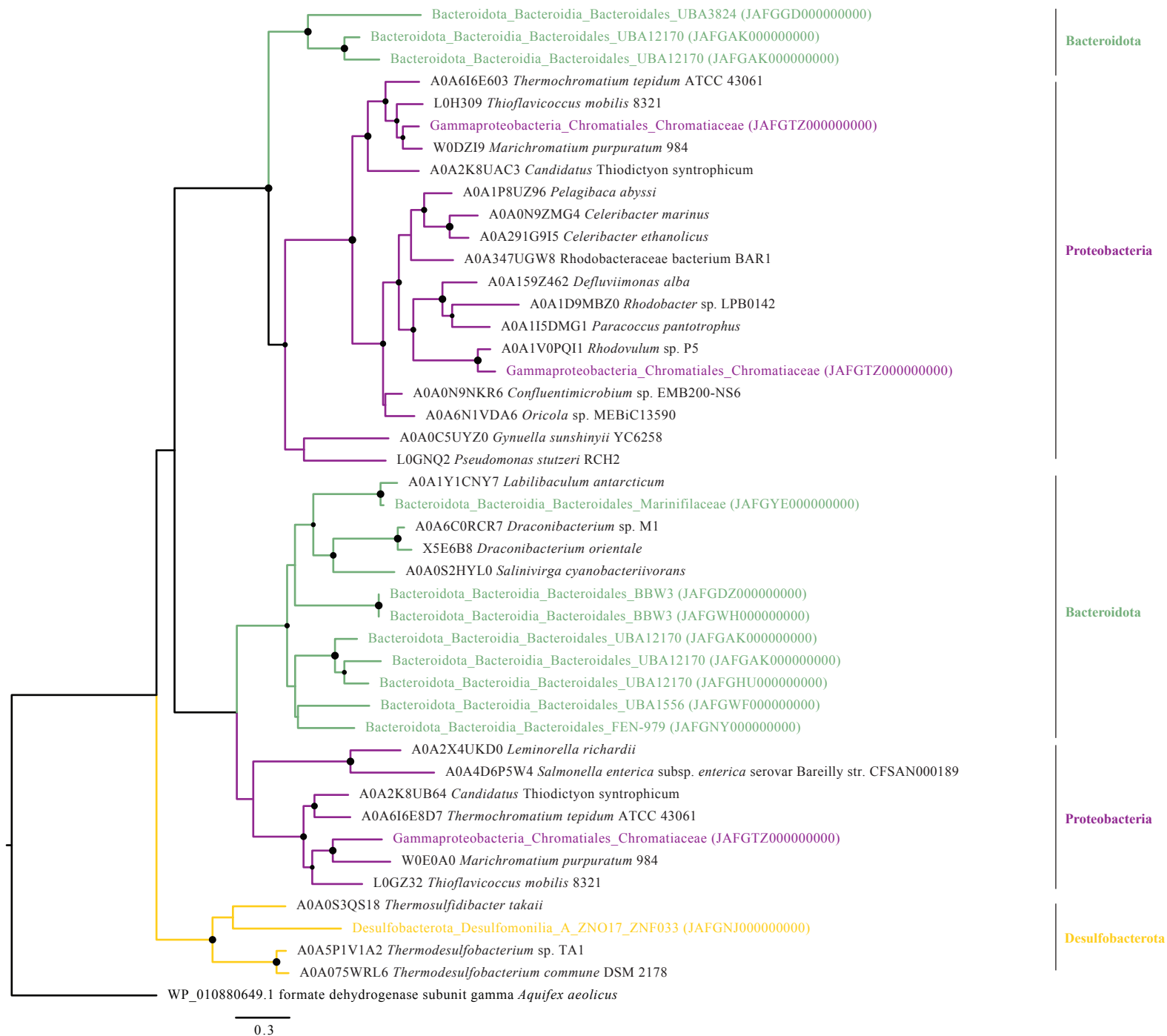

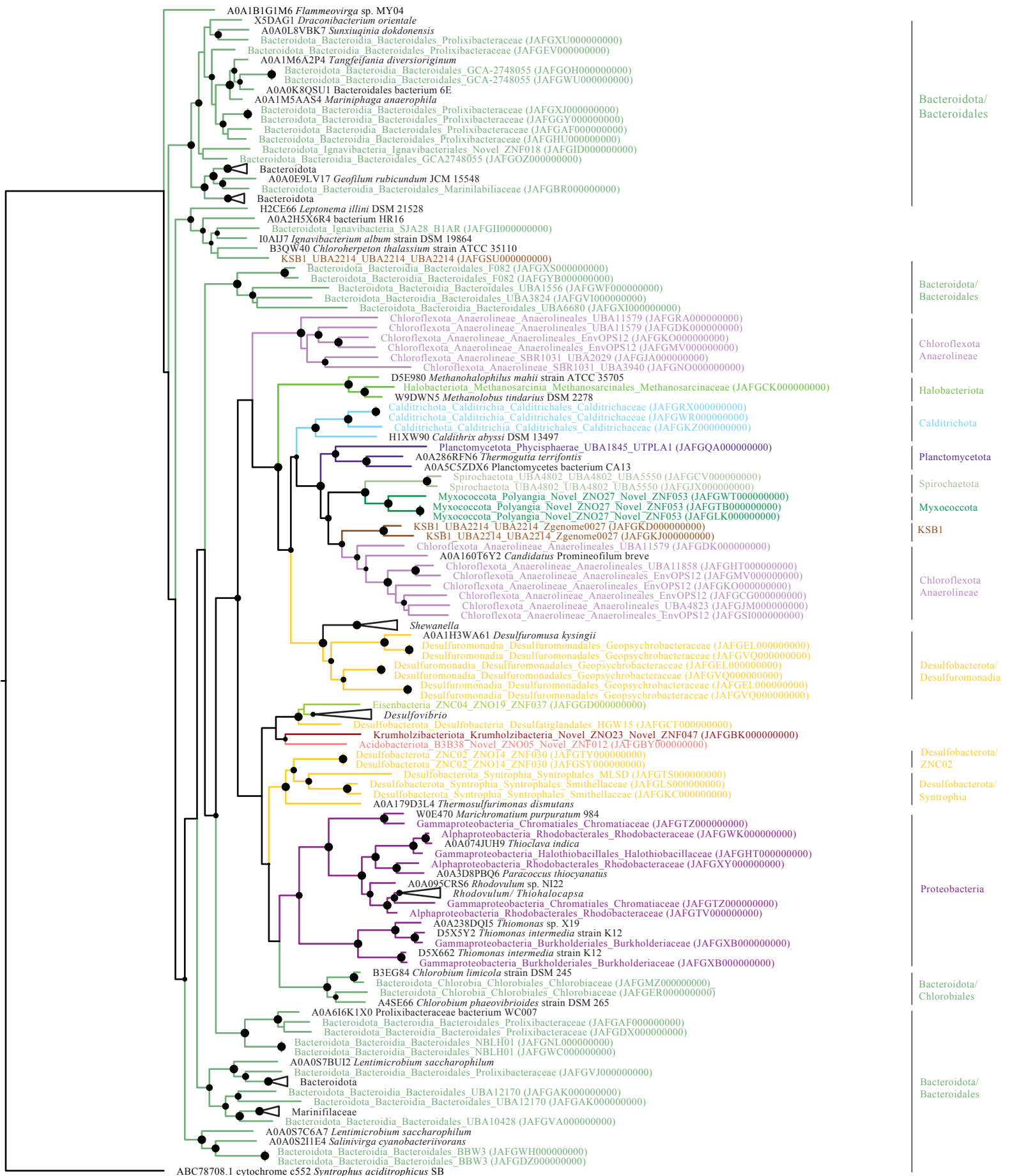

Supplement: FIG S6 [file mbio.00016-22-sf006.pdf]
