# Supplementary material for: Microbial Diversity and Sulfur Cycling in an Early Earth Analogue: From Ancient Novelty to Modern Commonality
Source: mBio. 2022 Mar 8;13(2):e00016-22. doi: 10.1128/mbio.00016-22 (PMC9040765; doi:10.1128/mbio.00016-22)
Supplement: FIG S2 [file mbio.00016-22-sf002.pdf]

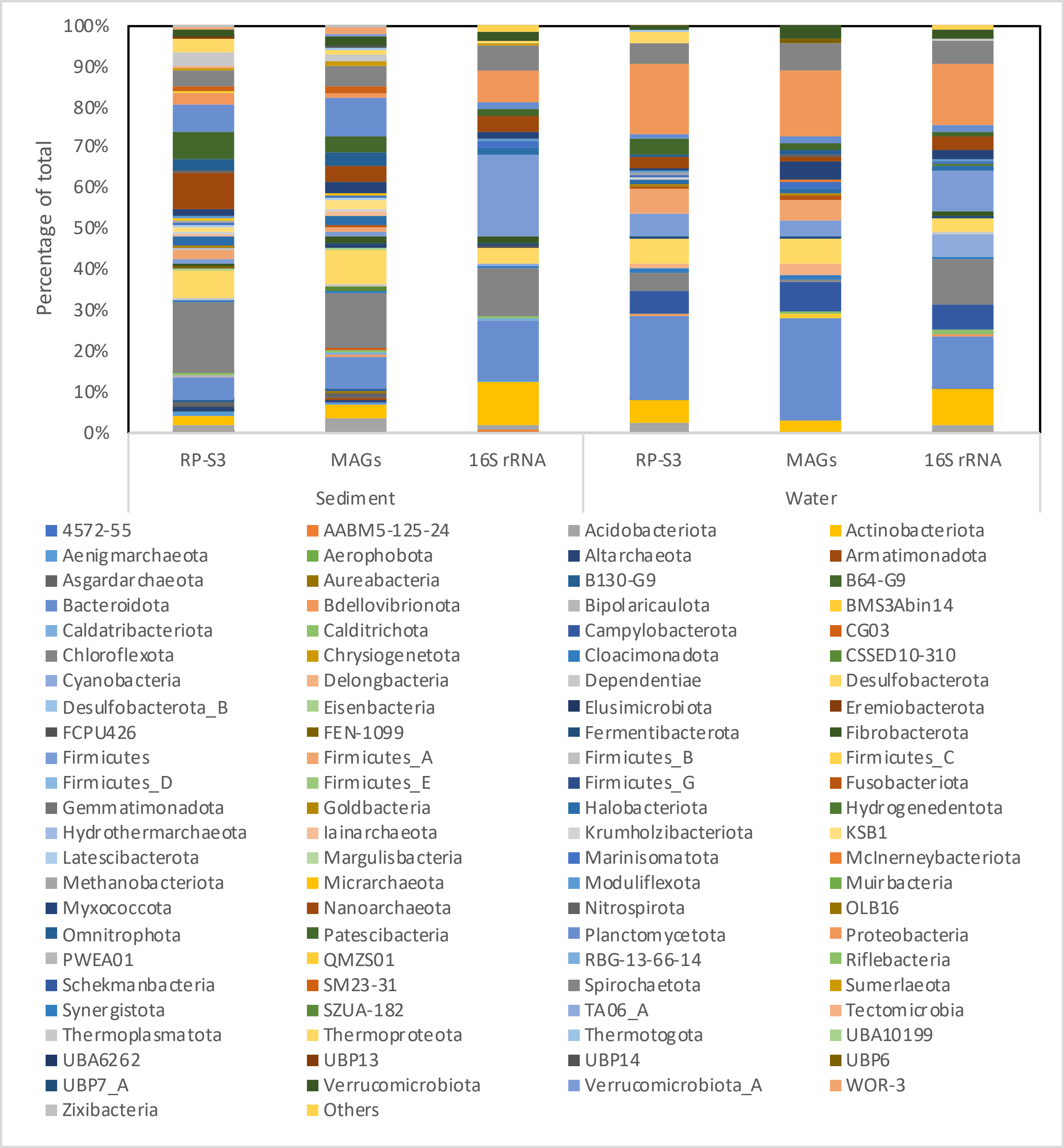

100%
90%
80%
70%
60%
50%
40%
30%
20%
10%
0%
Percentage of total
RP-S3
MAGs
16S rRNA
Sediment
RP-S3
MAGs
16S rRNA
Water
4572-55
AABM5-125-24
Acidobacteriota
Actinobacteriota
Aenigmarchaeota
Aerophobota
Altarchaeota
Armatimonadota
Asgardarchaeota
Aureabacteria
B130-G9
B64-G9
Bacteroidota
Bdellovibrionota
Bipolaricaulota
BMS3Abin14
Caldatribacteriota
Calditrichota
Campylobacterota
CG03
Chloroflexota
Chrysiogenetota
Cloacimonadota
CSSED10-310
Cyanobacteria
Delongbacteria
Dependentiae
Desulfobacterota
Desulfobacterota_B
Eisenbacteria
Elusimicrobiota
Eremiobacterota
FCPU426
FEN-1099
Fermentibacterota
Fibrobacterota
Firmicutes
Firmicutes_A
Firmicutes_B
Firmicutes_C
Firmicutes_D
Firmicutes_E
Firmicutes_G
Fusobacteriota
Gemmatimonadota
Goldbacteria
Halobacteriota
Hydrogenedentota
Hydrothermarchaeota
Iainarchaeota
Krumholzibacteriota
KSB1
Latescibacterota
Margulisbacteria
Marinisomatota
McInerneybacteriota
Methanobacteriota
Micrarchaeota
Moduliflexota
Muirbacteria
Myxococcota
Nanoarchaeota
Nitrospirota
OLB16
Omnitrophota
Patescibacteria
Planctomycetota
Proteobacteria
PWEA01
QMZS01
RBG-13-66-14
Riflebacteria
Schekmanbacteria
SM23-31
Spirochaetota
Sumerlaeota
Synergistota
SZUA-182
TA06_A
Tectomicrobia
Thermoplasmatota
Thermoproteota
Thermotogota
UBA10199
UBA6262
UBP13
UBP14
UBP6
UBP7_A
Verrucomicrobiota
Verrucomicrobiota_A
WOR-3
Zixibacteria
Others
