# Supplementary material for: Microbial Diversity and Sulfur Cycling in an Early Earth Analogue: From Ancient Novelty to Modern Commonality
Source: mBio. 2022 Mar 8;13(2):e00016-22. doi: 10.1128/mbio.00016-22 (PMC9040765; doi:10.1128/mbio.00016-22)
Supplement: TEXT S1 [file mbio.00016-22-t0001.docx]

**Supplementary document for**

**Microbial diversity and sulfur cycling in an early earth analogue: From ancient novelty to modern commonality**

**C. Ryan Hahn**^1^**, Ibrahim F. Farag**^1$^**, Chelsea L. Murphy**^1^**, Mircea Podar**^2,3^**, Mostafa S. Elshahed**^1^**, and Noha H. Youssef**^1^*****

**Supplementary Text**

**I. Methods**

**Analysis of sulfur cycling genes.**

*Sulfate-reduction.* Sulfate reduction capacity was assessed by the presence of genes encoding the enzymes 3'-phosphoadenosine 5'-phosphosulfate synthase [Sat; EC:2.7.7.4 2.7.1.25] for sulfate activation to adenylyl sulfate (APS), the enzyme complex adenylylsulfate reductase [AprAB; EC:1.8.99.2] for APS reduction to sulfite, the quinone-interacting membrane-bound oxidoreductase complex [QmoABC] for electron transfer, the enzyme dissimilatory sulfite reductase [DsrAB; EC:1.8.99.5] and its co-substrate DsrC for dissimilatory sulfite reduction to sulfide, and the sulfite reduction-associated membrane complex DsrMKJOP for linking cytoplasmic sulfite reduction to energy conservation.

*Sulfite-reduction.* Sulfite could be utilized by most sulfate-reducing microorganisms ^1^. Dedicated sulfite-reduction capacity was assessed by the presence of the dissimilatory sulfite reductase system explained above ^2, 3^ with the lack of sulfate-activation (Sat) and reduction (Apr) genes. In addition, sulfite-reduction was assessed via the sole or co-occurrence of the anaerobic sulfite reductase (AsrABC) system ^4^, along with the membrane-bound associated complex (HdrABC) for transfer of electrons to the AsrC subunit ^5^. The Asr enzyme has been shown to function in the cytoplasm in *Salmonella typhimurium* to reduce the sulfite released from respiratory reduction of tetrathionate and thiosulfate ^4^. However, a scenario where the Asr enzyme is involved in sulfite respiration is possible via electron transfer from a membrane-bound associated complex to AsrC (the physiological partner of AsrAB). A plausible candidate for this membrane complex is the heterodisulfide reductase-related enzymes (HdrABC), analogous to what was suggested for DsrC (the physiological partner of DsrAB) in organisms lacking the sulfite reduction-associated membrane complex DsrMKJOP ^5^.

*Polysulfide reduction:* In addition to sulfate and sulfite, Zodletone spring is euxinic with extremely high levels of zero valent sulfur, available as soluble polysulfide. Respiratory polysulfide reduction was assessed via the identification of the membrane-bound molybdoenzyme complex PsrABC, which reduces polysulfides with electrons obtained from either a hydrogenase or a formate dehydrogenase through a quinone electron carrier ^6^. In addition to the membrane-bound Psr system, representatives of the cytolpasmic sulfurhydrogenase I (HydABCD system), and/or II (ShyABCD system) were identified. However, although these enzymes have been shown to be dissimilatory in the archaeon *Pyrococcus furiosus* ^7, 8^, their involvement in an ETS-associated respiration is currently unclear.

*Thiosulfate reduction/ disproportionation:* Thiosulfate occurs in natural environments as a result of the reaction of sulfite with bisulfide (HS^-^) ^9^. Thiosulfate is relatively stable at neutral pH and is present in high levels in Zodletone spring, Thiosulfate contains two sulfur atoms: a sulfone-sulfur (oxidation state +5), and a sulfane-sulfur (oxidation state -1). As such, thiosulfate can be disproportionated where the sulfone-sulfur is reduced (serves as an electron acceptor), and the sulfane-sulfur is oxidized (serves as an electron donor), with the products being hydrogen sulfide, and sulfite, respectively. We searched for genes encoding the three known pathways for thiosulfate-disproportionation. First, in pure cultures of several sulfate reducers in the Desulfobacterota and Firmicutes, e.g. *Desulfovibrio*, *Desulfotomaculum*, thiosulfate disproportionation is known to occur via a cytochrome c-dependent thiosulfate reductase [EC: 1.8.2.5] ^10, 11, 12, 13, 14, 15, 16, 17^. Second, in pure culture members of the family Enterobacteriaceae (Gammaproteobacteria), thiosulfate disproportionation is known to occur via the quinone-dependent membrane-bound molybdopterin-containing thiosulfate reductase PhsABC ^18^. Finally, thiosulfate disproportionation to sulfite and hydrogen sulfide can also occur via a rhodanase-like enzyme [EC: 2.8.1.1 or EC: 2.8.1.3], as shown for several bacterial lineages ^19, 20, 21, 22, 23^, although this could be part of a thiosulfate assimilatory pathway as recently shown in *E. coli* ^24^.

Following the disproportionation of thiosulfate to sulfite and hydrogen sulfide, microorganisms differ in the fate of the produced sulfite. Some microorganisms reduce the released sulfite to sulfide via a Dsr or Asr dissimilatory sulfite reductase ^18^), leading to complete reduction of one thiosulfate molecule to two sulfides (thiosulfate-reduction). Others oxidize the released sulfite to sulfate via the reversal of the sulfate reduction pathway ^17, 25^, or via the sulfite dehydrogenases SorAB or SoeABC ^26^, leading to the final conversion of one thiosulfate molecule to one sulfide and one sulfate molecules. The distribution of all thiosulfate disproportionation capacities was assessed by the occurrence of one of the three pathways described above, and the fate of sulfite in genomes mediating the initial disproportionation steps was assessed as described above.

*Tetrathionate reduction:* Tetrathionate has two sulfur atoms in oxidation state of 0 while the other two are in oxidation state of +5. In nature, tetrathionate is formed via the biotic or abiotic oxidation of thiosulfate under anoxic conditions ^9^. Some microorganisms are capable of tetrathionate respiration via membrane-bound tetrathionate reductases that will reduce tetrathionate to thiosulfate serving as the terminal oxidase in a short electron transport system. Enzymes mediating such process include octaheme tetrathionate reductase (otr) ^27^, as well as the guanylyl molybdenum cofactor-containing tetrathionate reductase (ttrABC) ^28^. The produced thiosulfate could be metabolized through disproportionation as described above.

*Oxidative sulfur processes.* The versatile sulfur oxidation (SOX enzyme complex) system was assessed in all genomes. The SOX system mediates the oxidation of a wide range of reduced sulfur compounds (sulfide, sulfite, thiosulfate, and elemental sulfur) directly to sulfate*.* Sulfide oxidation to sulfur was also assessed by the presence of the sulfide dehydrogenase FccAB [EC: 1.8.2.3] and/or the sulfide:quinone oxidoreductase Sqr [EC: 1.8.5.4], both known to oxidize sulfide to sulfur or polysulfide. Sulfur/polysulfide oxidation to sulfite was assessed via the reversal of the Dsr system (encompassing the full Dsr system *dsrAB*+*dsrC*+*dsrMKJOP*, in addition to the genes *dsrEFH*, *tusA*, and *rhdA*). Sulfite oxidation to sulfate was assessed via the reversal of AprAB+QmoABC system, the sulfite dehydrogenase (quinone) SoeABC [EC: 1.8.5.6], or the sulfite dehydrogenase (c-type cytochrome) SorAB [EC: 1.8.2.1]. Thiosulfate oxidation to tetrathionate was assessed via the thiosulfate dehydrogenase *tsdA* [EC: 1.8.2.2], or the thiosulfate dehydrogenase (quinone) *doxAD* [EC: 1.8.5.2]. Tetrathionate generated could be cleaved using tetrathionate hydrolase (*tetH*) ^29^ that is known to cleave tetrathionate to thiosulfate, sulfur, and sulfate, or converted to sulfite using the rDSR system.

**II. Results.**

**1. Detailed phylogenomic analysis of Zodletone spring sediments and water communities.**

***Anoxic sediments.***

*Overall sequencing overview and diversity patterns.* Metagenomic sequencing of the spring sediments yielded 281 Gbp, 79.54% of which assembled into 12 Gbp contigs, with 6.8 Gbp contigs longer than 1Kbp. 1,848 genomes were binned, 683 of which passed quality control criteria, and 516 remained after dereplication (Table S1). These MAGs represented 64 phyla or candidate phyla (53 bacterial and 11 archaeal), 127 classes, 198 orders, and 300 families (Figure 1a-b). Diversity assessment utilizing small subunit ribosomal protein S3 from assembled contigs (n=2079), as well as a complementary 16S rRNA illumina sequencing effort (n=309,074 amplicons), identified a higher number of taxa (82 phyla and 1679 species in the ribosomal protein S3 dataset, and 69 phyla and 1050 species in 16S rRNA dataset) (Figure S2). Nevertheless, the overall community composition profiles generated from all three approaches were broadly similar.

*Detailed phylogenetic analysis of Zodletone sediment MAGs.* The Chloroflexota (n=69), Planctomycetota (n=47), Bacteroidota (n= 43), Desulfobacterota (n= 43), Spirochaetota (n= 28 genomes), Patescibacteria (n=20 genomes), and the archaeal phylum Nanoarchaeota (n=21) were the most abundant phyla in Zodletone spring sediments, albeit representing only 52.52% of the total number of recovered genomes (Figure 1, S3). A notable absence, or extreme paucity, of genomes belonging to the Proteobacteria (6 genomes) and Firmicutes (12 genomes), the most successful taxa within current biomes ^30^, as well as the oxygen-generating oxycayanobacteria (0 genomes) were observed (Figure 1, S3). Within the Chloroflexota, 38/69 genomes belonged to 3 novel orders, 5 novel families, and multiple LRD orders (Thermoflexales, 4572-78, and UBA2777) and families (E44-bin32, Fen-1058, J111, RBG-13-53-26, RBG-16-64-43, UBA11579, UBA11858, UBA2029, UBA2162, UBA3940, UBA4811, UBA4823, UBA5620, UBA5760, and UBA6092) (Figure 3a). Within the Planctomycetota, 17/47 genomes belonged to 2 novel orders, 8 novel families, and multiple LRD orders (FEN-1346, SZUA-567, and UBA8890) and families (Fen-1342, SM23-30, UBA1845, UTPLA1, and UBA8108, Figure 3c). Within the Bacteroidota, 27/43 genomes belonged to 1 novel family, and multiple LRD families (FEN-979, GCA-2748055, NBLH01, UBA10428, UBA12170, UBA5072, UBA6680, and SZUA-365, Figure 3b). Within the Spirochaetota, 19/28 genomes belonged to one novel class, 2 novel orders, and 9 novel families, as well as multiple LRD families (ARS1246, Marispirochaetaceae, and RPPD01, Figure 3d). Within the Desulfobacterota (Figure 3e), 35/43 genomes belonging to 3 novel classes, 10 novel orders, and 7 novel families were identified, as well as multiple LRD families (B25-G16, BuS5, HGW-15, MLS-D, NaphS2, UBA2210, and UBA3084). Only 6/43 genomes belonged to the well-described families Desulfovibrionaceae, Geopsychrobacteraceae, Smithellaceae, and Syntrophaceae. Finally, an extremely diverse community of Patescibacteria (13 different orders, 3 of which belonging to LRD orders, and 14 different families, including 6 novel and 2 LRD families), and Nanoarchaeota (2 orders including the LRD order CG07-land), and 15 different families, including 5 novel and 10 LRD families) were identified in the spring sediments (Figure 3F). A similar pattern of high proportion of novel and LRD families was identified throughout all other lineages (Figure 1a). Therefore, in addition to expanding the number of novel lineages (classes, orders, and families), and greatly enriching available genomes in rare, poorly represented taxa, our results highlight the uniqueness and distinction of the microbial community thriving in Zodletone spring sediments, compared to all previously studied habitats on the current earth.

***Hypoxic waters*.**

*Overall sequencing overview and diversity patterns.* Metagenomic sequencing of the oxygen-exposed overlaying water column community yielded 323 Gbp, 80.07% of which assembled into 3.6 Gbp contigs with 3.1 Gbp contigs >1K. 883 genomes were binned, with only 114 remaining after dereplication. Of these, 62 belonged to shared families with the sediment community, and 52 were water specific.

*Detailed phylogenetic analysis of Zodletone water MAGs.* Water-specific genomes (n=52) mostly belonged to well-characterized microbial lineages, e.g. class Alphaproteobacteria (5 genomes belonging to Rhodobacteraceae and Rhodospirillaceae, and 3 belonging to the uncultured lineages NBLK01 and Rs-D84), Gammaproteobacteria (9 genomes belonging to the lineages Thiomicrospiraceae, Halothiobacillaceae, Acidithiobacillaceae, Burkholderiaceae, Chromatiaceae, and Methylothermaceae, and 1 belonging to the uncultured lineage UBA9339), phylum Camplylobacterota (8 genomes, belonging to the families Sulfurimonadaceae, Sulfurovaceae, and Sulfurospirillaceae), Firmicutes/Firmicutes_A (4 genomes, and 6 genomes belonging to the Bacilli, and Clostridia classes, respectively), and well described families in the phyla Bacteroidota (Flavobacteriaceae, Prolixibacteraceae, Paludibacteraceae, Marinilabiliaceae, Tannerellaceae, Marinifilaceae, Balneolaceae), Desulfobacterota (Families Geopsychrobacteraceae, Desulfuromonadaceae,), and Spriochaetota (Sphaerochaetaceae, Treponemataceae, Spirochaetaceae_B). Collectively, this demonstrates a pattern where the intrusion of oxygen is associated with a negative impact on novel and LRD lineages that are prevalent in the sediment, and the propagation of communities associated with well-characterized lineages within the bacterial tree of life.

**2. Reductive sulfur processes dominate Zodletone spring sediment communities.** A total of 149 genomes (28.9 % of all genomes), belonging to 32 phyla, 51 classes, 69 orders, and 97 families were involved in at least one reductive sulfur processes (Figure 4, S4, Table S2). By comparison, only 21 sediment genomes (4.06% of all genomes) encoded at least one sulfur oxidation pathway (Figure 4, S4, Table S2). The reductive sulfur-community in the spring exhibited two unique traits: First, a majority of genomes encoding such capacities belonged to novel (47 genomes) or LRD (66 genomes) lineages (Figure 4, S4), and second: sulfite-, polysulfide-, thiosulfate-, and tetrathionate reduction appears to be more prevalent than sulfate-reduction capacities in the sediment genomes.

*Sulfate reduction.* Sulfate-reduction capacity was observed in only 18 sediment genomes (Figure 4, S4), but exhibited a unique community composition, when compared to well-studied marine and terrestrial habitats ^31, 32, 33, 34^. Sulfate-reduction capacities were observed in mostly previously undescribed or LRD lineages within the Zixibacteria, Acidobacteriota (members of family UBA6911, equivalent to Acidobacteria group 18), Myxococcota, Bacteroidota, Planctomycetota, candidate phylum OLB16 (1 genome), as well as rare and novel lineages within the Desulfobacterota (Figure 4, S4, S5a, Table S2). Organization of the sulfate reduction genes differed between different phyla, with Myxococcota, Zixibacteria, OLB16, and Acidobacteriota genomes encoding all genes for sulfate activation and reduction, dissimilatory sulfite reduction, as well as energy conservation on one locus, while Desulfobacterota genomes encoded genes for sulfate activation and reduction as well as energy conservation on one locus with genes for dissimilatory sulfite reduction on another, as previously observed in cultured Desulfobacterota ^35, 36, 37^.

*Sulfite reduction.* Sulfite (but not sulfate) reduction via the DsrAB+DsrC+DsrKMJOP system was identified in only 8 genomes belonging to 7 families within the phyla Planctomycetes, Chloroflexota, Spirochaetota, and Desulfobacterota (Figure 4, S4, S5b, S6a, Table S2). Gene organization of the *dsr* locus in the above 9 genomes differed between different phyla, with Chloroflexota, and Spirochaetota genomes encoding all genes for dissimilatory sulfite reduction (dsrABC plus dsrKMJOP) on one locus, while Planctomycetota and Desulfobacterota genomes showed a split *dsr* locus with dsrABC on one locus and dsrMKJOP on another (Figure S6a).

On the other hand, sulfite-reduction capacity within Zodletone spring sediment solely via the Asr/Hdr system was rampant, being encountered in 104 genomes belonging to 28 phyla, 43 (8 novel and 9 LRD) classes, 56 (18 novel, and 12 LRD) orders, and 72 (31 novel and 25 LRD) families (Figure 4, S4, S6b, Table S2), with a gene organization of the *asr* locus adjacent to the *hdr* locus in the majority of genomes (Figure S6b). Asr-encoding genomes in the sediments included members of predominantly previously undescribed and LRD lineages within the Chloroflexota, Desulfobacterota, Planctomycetota, and Bacteroidota. The capacity was also rampant in the yet-uncultured bacterial phyla, many of which have fairly limited distribution on the current earth, e.g. the candidate phyla CSSED10-310, FCPU426, RBG-13-66-14, SM23-31, SZUA-182, UBP14, Aureabacteria, Sumerlaeota. Interestingly, all genomes belonging to the novel phylum Krumholzibacteriota, recently described from the spring sediment ^38^, encoded complete anaerobic sulfite reductase systems. Zodletone dissimilatory sulfite reductase (Fig S7a) and the anaerobic sulfite reductase (Fig S6b) sequences clustered with reference sequences from the same phylum, generally showing no evidence of LGT.

*Sulfur (polysulfide) reduction.* Twenty Zodletone sediment genomes encoded *psrABC* genes (Figure 4, S4, S5c, S6c, Table S2). These genomes belonged mostly to previously undescribed and LRD families within the phyla Bacteroidota, Desulfobacterota, Myxococota, Acidobacterota, Chloroflexota, and Campylobacterota (Figure S4). In addition, representatives of the cytoplasmic sulfurhydrogenase I (HydABCD system), and/or II (ShyABCD system) were identified in 119 Zodletone sediment genomes (Figure 4). However, as explained above, direct involvement of these enzymes in an ETS-associated respiration is not yet clear.

*Thiosulfate disproportionation* *and reduction.* The quinone-dependent membrane-bound molybdopterin-containing thiosulfate reductase PhsABC was encoded in 11 genomes belonging to 6 phyla, with Bacteroidota representing the major phsABC-encoding phylum (4 genomes) (Table S2, Figure S5d). Within these genomes, only two (a Chloroflexota family UBA6092 genome, and a Desulfatiglandales family HGW15 genome) also encoded a dissimilatory sulfite reductase (the Asr system) akin to the Gammaproteobacteria thiosulfate disproportionating pure culture members, where the final products of thiosulfate disproportionation are expected to be only hydrogen sulfide (Figure 4, S4, S5d, Table S2). On the other hand, 5 of the 11 phsABC-encoding Zodletone genomes also encoded the sulfite dehydrogenase SoeABC system, akin to Desulfobacterota and Firmicutes pure culture members, where the final products of thiosulfate disproportionation are expected to be both hydrogen sulfide and sulfate (Figure 4, S4, S5d, Table S2). In addition to the phsABC system, 14 Zodletone genomes belonging to 6 phyla (Desulfobacterota,Acidobacteriota, Chloroflexota, Bacteroidota, Spirochaetota, and Myxococcota) encoded a rhodanase-like enzyme [EC: 2.8.1.1 or EC: 2.8.1.3] for thiosulfate disproportionation, as well as enzymes for both sulfite oxidation (by means of reversal of sulfate reduction via Sat+AprAB, or the sulfite dehydrogenase SoeABC), and sulfite reduction (via the dissimilatory sulfite reductases Dsr or Asr), where the final products of thiosulfate disproportionation are expected to be both hydrogen sulfide and sulfate (Figure 4, S4, S5d, Table S2).

*Tetrathionate reduction.* Seventy-three Zodletone sediment genomes encoded the octaheme tetrathionate reductase (OTR) enzyme. These genomes belonged to 14 phyla with major contribution from Bacteroidota (30 genomes), Chloroflexota (10 genomes), and Desulfobacterota (10 genomes) (Table S2, Figure 4, S4, S6e). In addition to Otr, 68 Zodletone genomes encoded the Ttr enzyme system. These genomes belonged to 14 phyla with major contribution from Chloroflexota (22 genomes), and Desulfobacterota (20 genomes) (Table S2, Figure 4, S4, S5e). As shown previously in *Salmonella typhimurium* ^39^, in presence of means for thiosulfate disproportionation/reduction and sulfite reduction, the thiosulfate produced as a result of tetrathionate reduction could be further reduced to sulfide. Out of the 105 sediment genomes encoding the Otr, and/or Ttr enzymes, only 12 genomes also encoded thiosulfate and sulfite reduction enzymes. These genomes belonged to the phyla Acidobacteriota, Chloroflexota, Desulfobacterota, Myxococcota, and Spirochaetota (Table S2).

*Substrates supporting sulfidogenic capacities at Zodletone spring.* Within lineages mediating reductive sulfur processes in Zodletone sediments (n=98), a wide range of substrates supporting sulfidogenesis were identified (Table S3, Figure S4). These included hexoses (26-87% of sulfidogenic lineages), pentoses (30-41% of sulfidogenic lineages), amino acids and peptides (39% of lineages), short chain fatty acids, e.g. lactate, propionate, butyrate, and acetate (22-73% of lineages), long chain fatty acids (29% of lineages), aromatic hydrocarbons (3% of lineages), and short chain alkanes (6% of lineages). Autotrophic capacities with hydrogen as the electron donor were identified in 28% of sulfidogenic lineages.

**3. Transcriptomic analysis.** Transcriptional expression of genes involved in S-species reduction/disproportionation was analyzed, and the identity of the active sulfur-reducing community in the spring sediment was examined (Figure 4). All S-species reduction/ disproportionation genes discussed above were identified in the metatranscriptomic dataset, and transcripts belonging to 51 different phyla were identified. Analysis of the spring sediment revealed the transcription of both the Dsr and Asr systems for sulfite reduction with contributions from Chloroflexota, Planctomycetota, Desulfobacterota, Bacteroidota, Fermentibacterota, Acidobacteriota, CSSED10-310, Actinobacteriota, Spirochaetota, WOR-3, and Fibrobacterota (Asr), and Desulfobacterota, Acidobacteriota, Zixibacteria, and Myxococcota (Dsr). Sulfate reduction genes (Sat, AprAB, and QmoABC) were also transcribed with major contribution from Desulfobacterota, Myxococcota, Zixibacteria, and Acidobacteriota. Total transcription levels of the Asr system were 4-times higher than the Dsr system, consistent with the higher number of Zodletone sediment genomes encoding the Asr system compared to the Dsr system. Transcription of the thiosulfate disproportionating rhodanese-like enzyme [EC: 2.8.1.1 or EC: 2.8.1.3] was detected in the phyla Desulfobacterota, Actinobacteriota, Firmicutes_A, Chloroflexota, Fibrobacterota, Planctomycetota, Spirochaetota, Bacteroidota, Halobacteriota, and Acidobacteriota, while the transcription of the thiosulfate reductase *phsABC* was detected in the phyla Actinobacteriota and Bacteroidota. Transcription of the tetrathionate reduction genes *ttrABC* was detected in the phyla Desulfobacterota, Actinobacteriota, Bacteroidota, Chloroflexota, Acidobacteriota, and Spirochaetota, while the octaheme tetrathionate reductase *otr* transcription was detected in Desulfobacterota, Bacteroidota, Myxococcota, UBP7_A, and Chloroflexota. Finally, the transcription of *psrABC* for polysulfide reduction was detected majorly in the phyla Bacteroidota, Desulfobacterota, and Campylobacterota, while transcription of the cytoplasmic sulfurhydrogenases I and II (*hyd/shy* systems) was identified in the phyla Actinobacteriota, Chloroflexota, Planctomycetota, Myxococcota, Acidobacteriota, Bacteroidota, and Desulfobacterota.

**4.** **Oxidative sulfur processes dominate Zodletone water community.** In contrast to sediment communities, reductive sulfur-processes were identified in only 25 (21.92%) water genomes, as opposed to 149 (29%) sediment genomes (Figure 4, S4, Table S2). Dissimilatory sulfate reduction to sulfide capacity was completely absent in water genomes. The capacity for dissimilatory sulfite reduction via the Dsr system was absent, and the Asr system was only encoded in 7 water genomes. Thiosulfate reduction/disproportionation capacity to sulfide and sulfate (PhsABC + SoeABC, and/or Rhodanase + Dsr/Asr + SoeABC/SorAB) was encoded in only four genomes, all of which also encoded the capacity for tetrathionate reduction (via Otr and/or ttrABC). Finally, respiratory polysulfide reduction (via PsrABC) was encoded in 19 genomes. In all cases, the reductive sulfur community in water was a subset of the sediment community.

In contrast, oxidative sulfur processes dominated the water community, with pathways encoding sulfide, sulfur, thiosulfate, tetrathionate, and/or sulfite oxidation to sulfate present in 59/114 genomes (51.8% of all water genomes) belonging to 13 phyla, 16 classes, 25 orders, and 43 families. The oxidative sulfur community in the water belonged to mostly well-characterized lineages (Table S2, Figure 4, S4). Only 8 and 10 genomes involved in oxidative sulfur processes belonged to novel, and LDR families, respectively.

A complete SOX system, putatively mediating oxidation of a wide range of reduced sulfur-species to sulfate was encoded in genomes belonging to well-characterized families within the Proteobacteria (11 genomes total belonging to families Acidithiobacillaceae, Burkholderiaceae, Halothiobacillaceae, Rhodobacteraceae, and Thiomicrospiraceae) and Campylobacterota (3 genomes in the family Sulfurimonadaceae) (Table S2, Figure 4, S4).

*Sulfide oxidation to sulfur and sulfite.* Thirty nine water genomes encoded the sulfide dehydrogenase fccAB [EC: 1.8.2.3] and/or the sulfide:quinone oxidoreductase Sqr [EC: 1.8.5.4] both known to oxidize sulfide to sulfur/ polysulfide (Table S2, Figure 4, S4). These genomes belonged to the phyla Bacteroidota (14 genomes in the well characterized families Chlorobiaceae, Prolixibacteraceae, and Paludibacteracaeae, as well as the uncultured families NBLH01, UBA1556, DTU049, and F082 in the order Bacteriodales), Proteobacteria (13 genomes in the families Acidithiobacillaceae, Burkholderiaceae, Chromatiaceae, Halothiobacillaceae, Methylothermaceae, Rhodobacteraceae, Thiomicrospiraceae), Campylobacterota (8 genomes in the families Sulfurimonadaceae, Sulfurospirillaceae, Sulfurovaceae), in addition to three genomes in the families Anaerolineaceae, Geopsychrobacteraceae, and UBA2242 within the phyla Chloroflexota, Desulfobacterota, and Marinisomatota, respectively, and one genome belonging to a novel Thermodesulfovibrionales family (Nitrospirota). Only two of the above thirty-nine genomes (one Proteobacteria genome and one Nitrospirota genome) encoded the capacity to further oxidize the sulfur/polysulfide to sulfite via the reversal of the Dsr system (encompassing the full Dsr system *dsrAB*+*dsrC*+*dsrMKJOP*, in addition to the genes *dsrEFH*, *tusA*, and *rhdA*).

*Sulfite oxidation to sulfate*: A total of twenty-six water genomes encoded the capacity for sulfite oxidation to sulfate via the reversal of AprAB+QmoABC system (1 Bacteroidales genome), the sulfite dehydrogenase (quinone) SoeABC [EC: 1.8.5.6] (22 genomes belonging to the order Bacteroidales, and the families Acidithiobacillaceae, Burkholderiaceae, Chromatiaceae, Dethiosulfatibacteraceae, Halothiobacillaceae, Methylothermaceae, Rhodobacteraceae, Thiomicrospiraceae within Proteobacteria, the families Sulfurimonadaceae, Sulfurospirillaceae, Sulfurovaceae within Campylobacterota, the Syntrophales family UBA3084, and a novel Thermodesulfovibrionales family (Nitrospirota)), or the sulfite dehydrogenase (cytochrome) SorAB [EC: 1.8.2.1] (3 genomes total within the families Chromatiaceae, Halothiobacillaceae (Proteobacteria), and UBA12059 (Spirochaetota)) (Table S2, Figure 4, S4).

*Thiosulfate oxidation to tetrathionate, and complete thiosulfate oxidation to sulfate via tetrathionate*: Eight water genomes encoded thiosulfate to tetrathionate oxidation capacities via either the thiosulfate dehydrogenase *tsdA* [EC: 1.8.2.2] (7 genomes belonging to the families Sulfurimonadaceae within Campylobacterota, and Rhodobacteraceae, Burkholderiaceae, Halothiobacillaceae, Thiomicrospiraceae within Proteobacteria), or the thiosulfate dehydrogenase (quinone) *doxAD* [EC: 1.8.5.2] (1 Flavobacteriaceae genome) (Table S2, Figure 4, S4). Two of these 8 genomes (1 Rhodobacteraceae, and 1 Halothiobacillaceae genomes) also encoded tetrathionate hydrolase (*tetH*) ^29^ that is known to cleave tetrathionate to thiosulfate, sulfur, and sulfate (Table S2, Figure 4, S4). Simultaneous identification of the SOX system and both forms of sulfide dehydrogenase (fccAB and Sqr) imply that these two genomes encode the capacity for complete thiosulfate oxidation to sulfate.

*Tetrathionate oxidation*: In addition to the above two genomes, ten other water genomes encoded tetrathionate hydrolase, but with no other means of thiosulfate and sulfide oxidation capacities (Table S2, Figure 4, S4). Surprisingly, tetH (without other means of thiosulfate or sulfide oxidation) was also encoded in 100 sediment genomes, belonging to 26 phyla, 37 classes, 49 orders, and 66 families (including 23 novel families). Only nine of these genomes (belonging to 9 families including 4 novel ones) showed *tetH* transcriptional levels above 1 (Table S2, Figure 4, S4). However, the exact function of tetrathionate hydrolase in these organisms is not entirely clear, as the subsequent steps of oxidation could not be identified.

**5. Additional metabolic capacities in Zodletone spring sediments.** In addition to reductive sulfur processes, strict fermentative capacities were highly prevalent in sediment genomes (Table S3), being identified in 100 of the 291 lineages studied. On the other hand, a dearth of aerobic (only 38 lineages), nitrate (only 65 lineages encoded dissimilatory nitrite reduction to ammonium, with 2 of which also encoding the suite of genes for denitrification), Fe^3+^ respiration (8 lineages), or chemolithotrophic nitrifying (only 1 lineage encoded the combination of ammonia monooxygenase and hydroxylamine dehydrogenase), and photosynthetic capacities were identified (Figure 1a, Table S3). Strict fermentative lineages mediate the degradation a wide range of substrates, e.g. sugars (89 of the 100 fermentative lineages), amino acids (85 of the 100 fermentative lineages), short chain fatty acids (37 of the 100 fermentative lineages), complex carbohydrates (36 of the 100 fermentative lineages), long chain fatty acid oxidation (2 lineages), and short chain alkanes (1 lineage) (Table S3), producing a wide range of fermentative end products including lactate, formate, acetate, ethanol, succinate, and hydrogen. Primary productivity in the spring sediments appears to be mostly mediated via hydrogen utilization coupled to either sulfur-cycle intermediates reduction (27 lineages, Table S3), or to CO_2_ fixation by hydrogenotrophic methanogens and acetogens using the Wood-Ljungdahl pathway (8 lineages, Table S3).

**Supplementary Figures:**

**Figure S1. Zodletone spring source sediments and overlaid water.**

**Figure S2.** Zodletone spring phylum-level community composition based on ribosomal protein S3 (RP-S3), binned genomes (MAGs), as well as the gene for 16S rRNA for both the sediment and the water samples.

**Figure S3.** Phylum-level affiliation for sediment versus water genomes. Number of genomes belonging to each phylum is shown for the sediment (blue bars on the left) and the water (orange bars on the right).

**Figure S4.** Family-level distribution of the genomes involved in S cycling in the spring. The maximum likelihood tree was constructed in FastTree ^40^ based on the concatenated alignments of 120 single-copy genes obtained from Gtdb-TK ^41^. The branches represent family-level taxonomy and are color coded by phylum. For phyla with 2 families or less involved in S cycling, branches are labeled as Phylum_Class_Order_Family. For phyla where 3 or more families are involved in S cycling, the phylum is shown at the base of the colored wedge and the branches are labeled as Class_Order_Family. Lineages staring with ZN depict novel lineages as follows: ZNC, novel class; ZNO, novel order; and ZNF, novel family. Bootstrap support values are shown as bubbles for nodes with >70% support. Tracks around the tree represent (from innermost to outermost): heatmap for the number of genomes in each family, abundance in Gtdb based on the number of available genomes (abundant with more than 5 genomes, rare with 5 genomes or less, and novel with no genomes in Gtdb), pie charts of the breakdown of the number of genomes in the sediment (cyan) versus water (magenta), sulfur reduction pathways (5 tracks in purple), thiosulfate disproportionation pathways (1 track in golden brown), sulfur oxidation pathways (7 tracks in red), and substrates predicted to support growth depicted by colored stars (cyan, sugars; lime green, complex carbohydrates; magenta, amino acids; orange, proteins; purple, CO2 fixation; red, short-chain fatty acids (SCFA); blue, beta oxidation of long-chain fatty acids; brown, anaerobic benzoate/aromatic hydrocarbon degradation; black, anaerobic alkane degradation; and grey, Hydrogen oxidation).

**Figure S5.** Expanding lineages mediating reductive sulfur cycling processes in the tree of life. Annotree ^42^ was queried at the family level for the presence of **(A)** sulfate reduction genes (combined search for the genes AprA, AprB, Sat, QmoA, QmoB, QmoC, DsrA, DsrB, and DsrC), **(B)** sulfite reduction genes using both the DSR system (combined search for the genes DsrA, DsrB, and DsrC, excluding all duplicate hits from (A) and excluding all hits from phyla known to use the system in the oxidative direction (Proteobacteria, Nitrospira, and Chlorobiaceae), and the ASR system (combined search for the genes AsrA, AsrB, AsrC, HdrA, HdrB, and HdrC), **(C)** polysulfide reduction genes (combined search for the genes PsrA, PsrB, and PsrC), **(D)** thiosulfate reduction (combined search for the genes PhsA, PhsB, and PhsC, combined with either DsrA, DsrB, and DsrC , or AsrA, AsrB, and AsrC) and thiosulfate disproportionation (combined search for the genes PhsA, PhsB, and PhsC, combined with either AprA, AprB, and Sat, or SoeA, SoeB, and SoeC, or SorA), and **(E)** tetrathionate reduction (combined search for the genes TtrA, TtrB, and TtrC). For each of these searches, all hits were downloaded and sorted and parsed to keep one representative from each family with a hit. The Gtdb accession IDs for all such representatives were used to extract their 120 single-copy-protein concatenated alignment available from the Gtdb downloads repository (<https://data.gtdb.ecogenomic.org/releases/release95/95.0/genomic_files_reps/>). These were then combined with the concatenated alignments for family-level representatives in Zodletone with genomic evidence of the corresponding sulfur reductive. The maximum likelihood trees were constructed in FastTree ^40^. The branches represent family-level taxonomy and are color coded by phylum. Zodletone lineages staring with ZN depict novel lineages as follows: ZNC, novel class; ZNO, novel order; and ZNF, novel family. The track around the tree represents the distribution of each family level representative as follows: only encountered in Annotree, black; only encountered in Zodletone spring sediment, blue; and encountered in both, red. By comparing the number of lineages with the blue track (only encountered in Zodletone) to the combined number of lineages with the black and red tracks, the level of expansion of lineages mediating reductive sulfur cycling processes in the tree of life can be deduced as follows: with regards to sulfate reduction (A), and as stated in the results section of the main text, lineages encountered in Zodletone spring sediment encoded minimal sulfate reduction capacities. Only 7 new lineages were added to an existing list of 90 lineages in the bacteria tree of life (increase by 7.7%). On the other hand, with regards to sulfite reduction (B), representatives of 71 new families were added to a list of 76 families already known to encode this function (increase by 93.4%). For polysulfide reduction (C), thiosulfate reduction/disproportionation (D), and tetrathionate reduction (D), increases of 11.8% (12 new lineages added to 102 already known), 30.4% (7 new lineages added to 23 already known), and 34.7% (43 new lineages added to 124 already known), respectively, were encountered.

**Figure S6.** Phylogenetic affiliation and contig organization of selected sulfur reduction proteins. Phylogeny of the dissimilatory sulfite reductase DsrAB [EC:1.8.99.5] concatenated proteins **(A)**, anaerobic sulfite reductase subunit B AsrB **(B)**, polysulfide reductase subunit gamma PsrC **(C)**, thiosulfate reductase cytochrome b subunit PhsC **(D)**, and octaheme tetrathionate reductase Otr **(E)**. Alignments were created in Mafft ^43^ and maximum likelihood trees were constructed in RaxML ^44^. Bootstrap support values are shown as bubbles for nodes with >50% support. Branches and branch labels are color coded by phylum for Zodletone sequences. Branch labels depict classification to family level followed by the NCBI genome accession number. Reference sequences are shown in black with the Uniprot accession numbers. Contig organizations of the DSR and ASR loci in selected Zodletone genomes are shown to the right of the trees in A and B. Genes are color coded as shown in the top right corner. Unrelated genes are shown by grey arrows. Gene maps were created in R using the package genoplotR ^45^. Phylum/class classification is depicted to the right of the trees in C-E.

**Supplementary tables:**

**Table S1:** List of all genomes analyzed in this study with their NCBI Assembly accession numbers, taxonomic classification, sequencing statistics, and general genomic features.

**Table S2:** S-cycling genes predicted in Zodletone genomes. Genes are shown in the table header and actual gene names are shown in the corresponding cells.

**Table S3:** Substrates potentially supporting growth, predicted fermentation end products, and energy conservation pathways predicted from genomic analysis.

**References**

1. Rabus R, Hansen TA, Widdel F. Dissimilatory Sulfate- and Sulfur-Reducing Prokaryotes. In: *The Prokaryotes: Prokaryotic Physiology and Biochemistry* (eds Rosenberg E, DeLong EF, Lory S, Stackebrandt E, Thompson F). Springer Berlin Heidelberg (2013).

2. Hausmann B*, et al.* Peatland Acidobacteria with a dissimilatory sulfur metabolism. *Isme j* **12**, 1729-1742 (2018).

3. Colman DR, Lindsay MR, Amenabar MJ, Fernandes-Martins MC, Roden ER, Boyd ES. Phylogenomic analysis of novel Diaforarchaea is consistent with sulfite but not sulfate reduction in volcanic environments on early Earth. *The ISME Journal* **14**, 1316-1331 (2020).

4. Huang CJ, Barrett EL. Sequence analysis and expression of the Salmonella typhimurium asr operon encoding production of hydrogen sulfide from sulfite. *J Bacteriol* **173**, 1544-1553 (1991).

5. Venceslau SS, Stockdreher Y, Dahl C, Pereira IA. The "bacterial heterodisulfide" DsrC is a key protein in dissimilatory sulfur metabolism. *Biochim Biophys Acta* **1837**, 1148-1164 (2014).

6. Dietrich W, Klimmek O. The function of methyl-menaquinone-6 and polysulfide reductase membrane anchor (PsrC) in polysulfide respiration of Wolinella succinogenes. *Eur J Biochem* **269**, 1086-1095 (2002).

7. Blumentals, II, Itoh M, Olson GJ, Kelly RM. Role of Polysulfides in Reduction of Elemental Sulfur by the Hyperthermophilic Archaebacterium Pyrococcus furiosus. *Appl Environ Microbiol* **56**, 1255-1262 (1990).

8. Ma K, Adams MW. Sulfide dehydrogenase from the hyperthermophilic archaeon Pyrococcus furiosus: a new multifunctional enzyme involved in the reduction of elemental sulfur. *J Bacteriol* **176**, 6509-6517 (1994).

9. Zopfi J, Ferdelman TG, Fossing H. Distribution and fate of sulfur intermediates - sulfite, tetrathionate, thiosulfate, and elemental sulfur in marine sediments. In: *In Sulfur Biogeochemistry – Past and Pre- sent.* (eds Amend JP, Edwards KJ, Lyons TW). Geological Society of America, (2004).

10. Bak F, Cypionka H. A novel type of energy metabolism involving fermentation of inorganic sulphur compounds. *Nature* **326**, 891-892 (1987).

11. Bak F, Pfennig N. Chemolithotrophic growth of Desulfovibrio sulfodismutans sp. nov. by disproportionation of inorganic sulfur compounds. *Archives of Microbiology* **147**, 184-189 (1987).

12. Finster K, Liesack W, Thamdrup B. Elemental sulfur and thiosulfate disproportionation by Desulfocapsa sulfoexigens sp. nov., a new anaerobic bacterium isolated from marine surface sediment. *Appl Environ Microbiol* **64**, 119-125 (1998).

13. Jackson BE, McInerney MJ. Thiosulfate disproportionation by Desulfotomaculum thermobenzoicum. *Appl Environ Microbiol* **66**, 3650-3653 (2000).

14. Janssen PH, Schuhmann A, Bak F, Liesack W. Disproportionation of inorganic sulfur compounds by the sulfate-reducing bacterium Desulfocapsa thiozymogenes gen. nov., sp. nov. *Archives of Microbiology* **166**, 184-192 (1996).

15. Jørgensen BB. A thiosulfate shunt in the sulfur cycle of marine sediments. *Science* **249**, 152-154 (1990).

16. Jørgensen BB, Bak F. Pathways and microbiology of thiosulfate transformations and sulfate reduction in a marine sediment (kattegat, denmark). *Appl Environ Microbiol* **57**, 847-856 (1991).

17. Krämer M, Cypionka H. Sulfate formation via ATP sulfurylase in thiosulfate- and sulfite-disproportionating bacteria. *Archives of Microbiology* **151**, 232-237 (1989).

18. Heinzinger NK, Fujimoto SY, Clark MA, Moreno MS, Barrett EL. Sequence analysis of the phs operon in Salmonella typhimurium and the contribution of thiosulfate reduction to anaerobic energy metabolism. *J Bacteriol* **177**, 2813-2820 (1995).

19. Aird BA, Heinrikson RL, Westley J. Isolation and characterization of a prokaryotic sulfurtransferase. *J Biol Chem* **262**, 17327-17335 (1987).

20. Etchebehere C, Muxí L. Thiosulfate reduction and alanine production in glucose fermentation by members of the genus Coprothermobacter. *Antonie Van Leeuwenhoek* **77**, 321-327 (2000).

21. Kaji A, Mc EW. Mechanism of hydrogen sulfide formation from thiosulfate. *J Bacteriol* **77**, 630-637 (1959).

22. Peck HD, Jr., Fisher E, Jr. The oxidation of thiosulfate and phosphorylation in extracts of Thiobacillus thioparus. *J Biol Chem* **237**, 190-197 (1962).

23. Ravot G*, et al.* Thiosulfate reduction, an important physiological feature shared by members of the order thermotogales. *Appl Environ Microbiol* **61**, 2053-2055 (1995).

24. Kawano Y*, et al.* Improved fermentative L-cysteine overproduction by enhancing a newly identified thiosulfate assimilation pathway in Escherichia coli. *Appl Microbiol Biotechnol* **101**, 6879-6889 (2017).

25. Finster K. Microbiological disproportionation of inorganic sulfur compounds. *Journal of Sulfur Chemistry* **29**, 281-292 (2008).

26. Frederiksen TM, Finster K. Sulfite-oxido-reductase is involved in the oxidation of sulfite in Desulfocapsa sulfoexigens during disproportionation of thiosulfate and elemental sulfur. *Biodegradation* **14**, 189-198 (2003).

27. Mowat CG*, et al.* Octaheme tetrathionate reductase is a respiratory enzyme with novel heme ligation. *Nat Struct Mol Biol* **11**, 1023-1024 (2004).

28. Hinojosa-Leon M, Dubourdieu M, Sanchez-Crispin JA, Chippaux M. Tetrathionate reductase of Salmonella thyphimurium: a molybdenum containing enzyme. *Biochem Biophys Res Commun* **136**, 577-581 (1986).

29. Kanao T, Kamimura K, Sugio T. Identification of a gene encoding a tetrathionate hydrolase in Acidithiobacillus ferrooxidans. *J Biotechnol* **132**, 16-22 (2007).

30. Nayfach S*, et al.* A genomic catalog of Earth's microbiomes. *Nat Biotechnol*, (2020).

31. Holmer M, Storkholm P. Sulphate reduction and sulphur cycling in lake sediments: a review. *Freshw Biol* **46**, 431-451 (2001).

32. Jørgensen BB, Findlay AJ, Pellerin A. The biogeochemical sulfur-cycle in marine sediments. *Front Microbiol* **10**, 849 (2019).

33. Vavourakis CD*, et al.* Metagenomes and metatranscriptomes shed new light on the microbial-mediated sulfur cycle in a Siberian soda lake. *BMC Microbiol* **17**, 69 (2019).

34. Wasmund K, Mußmann M, Loy A. The life sulfuric: microbial ecology of sulfur cycling in marine sediments. *Environ Microbiol Rep* **9**, 323-344 (2017).

35. Zecchin S*, et al.* Rice Paddy <span class="named-content genus-species" id="named-content-1">Nitrospirae</span> Carry and Express Genes Related to Sulfate Respiration: Proposal of the New Genus “<span class="named-content genus-species" id="named-content-2">Candidatus</span> Sulfobium”. *Applied and Environmental Microbiology* **84**, e02224-02217 (2018).

36. Venceslau SS, Stockdreher Y, Dahl C, Pereira IAC. The “bacterial heterodisulfide” DsrC is a key protein in dissimilatory sulfur metabolism. *Biochimica et Biophysica Acta (BBA) - Bioenergetics* **1837**, 1148-1164 (2014).

37. Mussmann M*, et al.* Clustered genes related to sulfate respiration in uncultured prokaryotes support the theory of their concomitant horizontal transfer. *J Bacteriol* **187**, 7126-7137 (2005).

38. Youssef NH*, et al.* Candidatus Krumholzibacterium zodletonense gen. nov., sp nov, the first representative of the candidate phylum Krumholzibacteriota phyl. nov. recovered from an anoxic sulfidic spring using genome resolved metagenomics. *Systematic and Applied Microbiology* **42**, 85-93 (2019).

39. Price-Carter M, Tingey J, Bobik TA, Roth JR. The alternative electron acceptor tetrathionate supports B12-dependent anaerobic growth of Salmonella enterica serovar typhimurium on ethanolamine or 1,2-propanediol. *J Bacteriol* **183**, 2463-2475 (2001).

40. Price MN, Dehal PS, Arkin AP. FastTree 2 – Approximately Maximum-Likelihood Trees for Large Alignments. *PLOS ONE* **5**, e9490 (2010).

41. Chaumeil P-A, Mussig AJ, Hugenholtz P, Parks DH. GTDB-Tk: a toolkit to classify genomes with the Genome Taxonomy Database. *Bioinformatics* **36**, 1925-1927 (2019).

42. Mendler K, Chen H, Parks DH, Lobb B, Hug LA, Doxey AC. AnnoTree: visualization and exploration of a functionally annotated microbial tree of life. *Nucleic acids research* **47**, 4442-4448 (2019).

43. Nakamura T, Yamada KD, Tomii K, Katoh K. Parallelization of MAFFT for large-scale multiple sequence alignments. *Bioinformatics* **34**, 2490-2492 (2018).

44. Stamatakis A. RAxML version 8: a tool for phylogenetic analysis and post-analysis of large phylogenies. *Bioinformatics* **30**, 1312-1313 (2014).

45. Guy L, Kultima JR, Andersson SGE. genoPlotR: comparative gene and genome visualization in R. *Bioinformatics (Oxford, England)* **26**, 2334-2335 (2010).
