# Supplementary material for: Microbial Diversity and Sulfur Cycling in an Early Earth Analogue: From Ancient Novelty to Modern Commonality
Source: mBio. 2022 Mar 8;13(2):e00016-22. doi: 10.1128/mbio.00016-22 (PMC9040765; doi:10.1128/mbio.00016-22)
Supplement: FIG S5 [file mbio.00016-22-sf005.pdf]

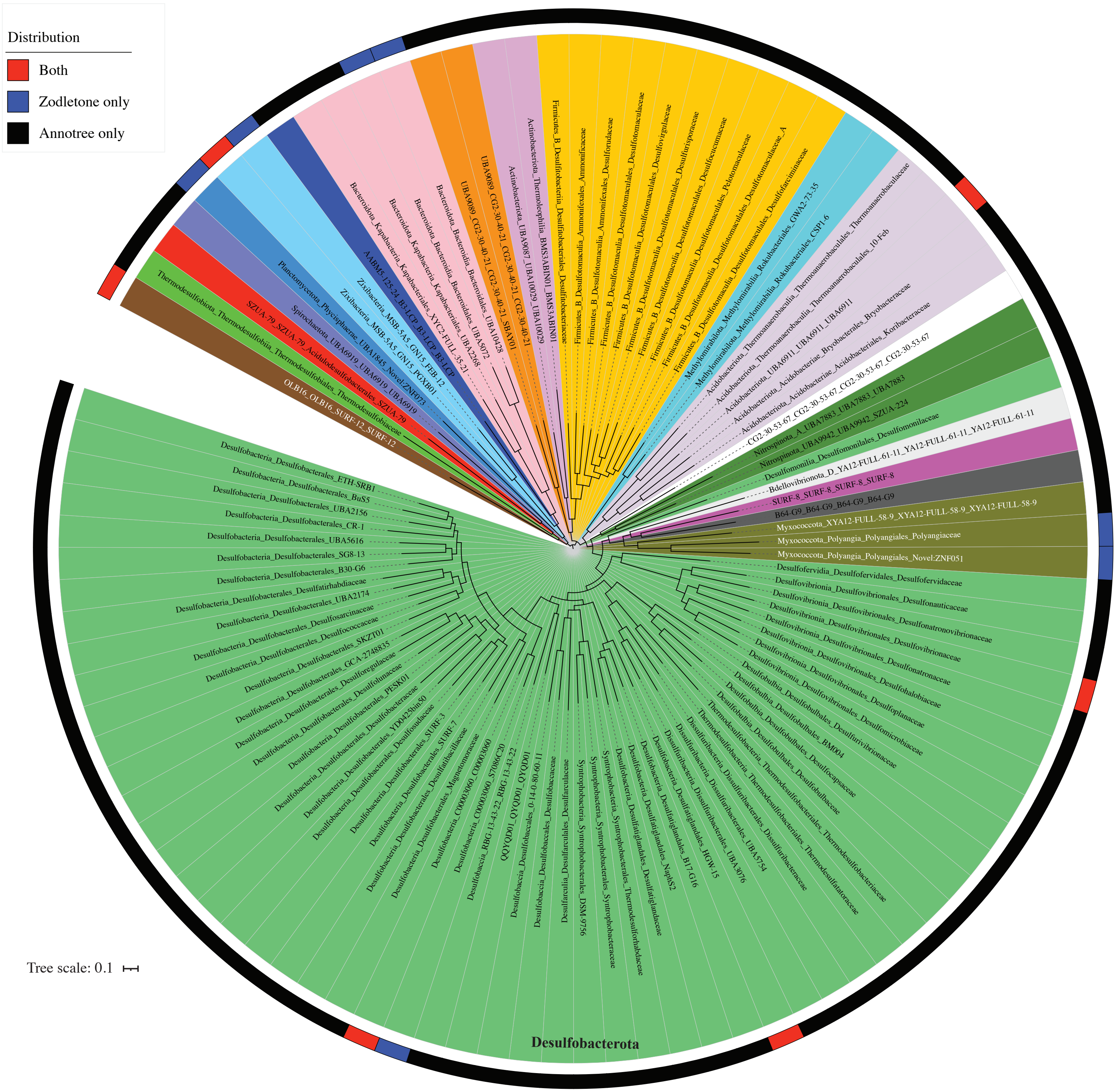
Distribution
Both
Zodletone only
Annotree only
Tree scale: 0.1
Desulfobacterota
Desulfobacteria_Desulfobacterales_ETH-SRB1
Desulfobacteria_Desulfobacterales_BuS5
Desulfobacteria_Desulfobacterales_UBA2156
Desulfobacteria_Desulfobacterales_CR-1
Desulfobacteria_Desulfobacterales_UBA5616
Desulfobacteria_Desulfobacterales_SG8-13
Desulfobacteria_Desulfobacterales_B30-G6
Desulfobacteria_Desulfobacterales_Desulfatirhabdiaceae
Desulfobacteria_Desulfobacterales_UBA2174
Desulfobacteria_Desulfobacterales_Desulfosarcinaceae
Desulfobacteria_Desulfobacterales_Desulfococcaceae
Desulfobacteria_Desulfobacterales_SKZT01
Desulfobacteria_Desulfobacterales_GCA-2748835
Desulfobacteria_Desulfobacterales_Desulforegulaceae
Desulfobacteria_Desulfobacterales_Desulfolunaceae
Desulfobacteria_Desulfobacterales_PESK01
Desulfobacteria_Desulfobacterales_Desulfobacteraceae
Desulfobacteria_Desulfobacterales_YD0425bin50
Desulfobacteria_Desulfobacterales_Desulfosudaceae
Desulfobacteria_Desulfobacterales_SURF-3
Desulfobacteria_Desulfobacterales_SURF-7
Desulfobacteria_Desulfobacterales_Desulfatibacillaceae
Desulfobacteria_Desulfobacterales_Magnetomoraceae
Desulfobacteria_C00003060_C00003060
Desulfobacteria_C00003060_S7086C20
Desulfobaccia_RBG-13-43-22_RBG-13-43-22
QYQD01_QYQD01_QYQD01
Desulfobaccia_Desulfobaccales_0-14-0-80-60-11
Desulfobaccia_Desulfobaccales_Desulfobaccaceae
Desulfarculia_Desulfarculales_Desulfarculaceae
Syntrophobacteria_Syntrophobacterales_DSM-9756
Syntrophobacteria_Syntrophobacterales_Syntrophobacteraceae
Syntrophobacteria_Syntrophobacterales_Thermodesulforhabdaceae
Desulfobacteria_Desulfatiglandales_Desulfatiglandaceae
Desulfobacteria_Desulfatiglandales_NaphS2
Desulfobacteria_Desulfatiglandales_B17-G16
Dissulfuribacteria_Dissulfuribacterales_HGW-15
Dissulfuribacteria_Dissulfuribacterales_UBA3076
Dissulfuribacteria_Dissulfuribacterales_UBA5754
Dissulfuribacteria_Dissulfuribacterales_Dissulfuribacteraceae
Thermodesulfobacteria_Thermodesulfobacteriales_Thermodesulfatatoraceae
Thermodesulfobacteria_Thermodesulfobacteriales_Thermodesulfobacteriaceae
Desulfobulbia_Desulfobulbales_Desulfobulbaceae
Desulfobulbia_Desulfobulbales_Desulfocapsaceae
Desulfobulbia_Desulfobulbales_BM004
Desulfovibrionia_Desulfovibrionales_Desulfurivibrionaceae
Desulfovibrionia_Desulfovibrionales_Desulfomicrobiaceae
Desulfovibrionia_Desulfovibrionales_Desulfoplanaceae
Desulfovibrionia_Desulfovibrionales_Desulfohalobiaceae
Desulfovibrionia_Desulfovibrionales_Desulfonatronaceae
Desulfovibrionia_Desulfovibrionales_Desulfovibrionaceae
Desulfovibrionia_Desulfovibrionales_Desulfonatronovibrionaceae
Desulfovibrionia_Desulfovibrionales_Desulfonauticaceae
Desulfofervidia_Desulfofervidales_Desulfofervidaceae
Myxococcota_Polyangia_Polyangiales_Novel:ZNF051
Myxococcota_Polyangia_Polyangiales_Polyangiaceae
Myxococcota_XYA12-FULL-58-9_XYA12-FULL-58-9_XYA12-FULL-58-9
B64-G9_B64-G9_B64-G9_B64-G9
SURF-8_SURF-8_SURF-8_SURF-8
Bdellovibrionota_D_YA12-FULL-61-11_YA12-FULL-61-11_YA12-FULL-61-11
Desulfomonilia_Desulfomonilales_Desulfomonilaceae
Nitrospinota_UBA9942_UBA9942_SZUA-224
Nitrospinota_A_UBA7883_UBA7883_UBA7883
CG2-30-53-67_CG2-30-53-67_CG2-30-53-67_CG2-30-53-67
Acidobacteriota_Acidobacteriae_Acidobacteriales_Koribacteraceae
Acidobacteriota_Acidobacteriae_Bryobacterales_Bryobacteraceae
Acidobacteriota_UBA6911_UBA6911_UBA6911
Acidobacteriota_Thermoanaerobaculia_Thermoanaerobaculales_10-Feb
Acidobacteriota_Thermoanaerobaculia_Thermoanaerobaculales_Thermoanaerobaculaceae
Methylomirabilota_Methylomirabilia_Rokubacteriales_CSP1-6
Methylomirabilota_Methylomirabilia_Rokubacteriales_GWA2-73-35
Firmicutes_B_Desulfotomaculia_Desulfotomaculales_Desulfofarciminaceae
Firmicutes_B_Desulfotomaculia_Desulfotomaculales_Desulfotomaculaceae_A
Firmicutes_B_Desulfotomaculia_Desulfotomaculales_Pelotomaculaceae
Firmicutes_B_Desulfotomaculia_Desulfotomaculales_Desulfocucumaceae
Firmicutes_B_Desulfotomaculia_Desulfotomaculales_Desulfitibacteraceae
Firmicutes_B_Desulfotomaculia_Desulfotomaculales_Desulfovirgulaceae
Firmicutes_B_Desulfotomaculia_Desulfotomaculales_Desulfotomaculaceae
Firmicutes_B_Desulfotomaculia_Ammonifexales_Desulforudaceae
Firmicutes_B_Desulfotomaculia_Ammonifexales_Ammonificaceae
Firmicutes_B_Desulfitobacteriia_Desulfitobacteriales_Desulfitobacteriaceae
Actinobacteriota_Thermoleophilia_BMS3ABIN01_BMS3ABIN01
Actinobacteriota_UBA9087_UBA10029_UBA10029
UBA9089_CG2-30-40-21_CG2-30-40-21_CG2-30-40-21
UBA9089_CG2-30-40-21_CG2-30-40-21_SBAY01
Bacteroidota_Bacteroidia_Bacteroidales_UBA10428
Bacteroidota_Bacteroidia_Bacteroidales_UBA5072
Bacteroidota_Kapabacteria_Kapabacteriales_UBA2268
Bacteroidota_Kapabacteria_Kapabacteriales_XYC2-FULL-35-21
AABM5-125-24_B3-LCP_B3-LCP_B3-LCP
Zixibacteria_MSB-5A5_GN15_FEB-12
Zixibacteria_MSB-5A5_GN15_PGXB01
Planctomycetota_Phycisphaerae_UBA1845_Novel:ZNF073
Spirochaetota_UBA6919_UBA6919_UBA6919
SZUA-79_SZUA-79_Acidulodesulfobacterales_SZUA-79
Thermodesulfobiota_Thermodesulfobiia_Thermodesulfobiales_Thermodesulfobiaceae
OLB16_OLB16_SURF-12_SURF-12

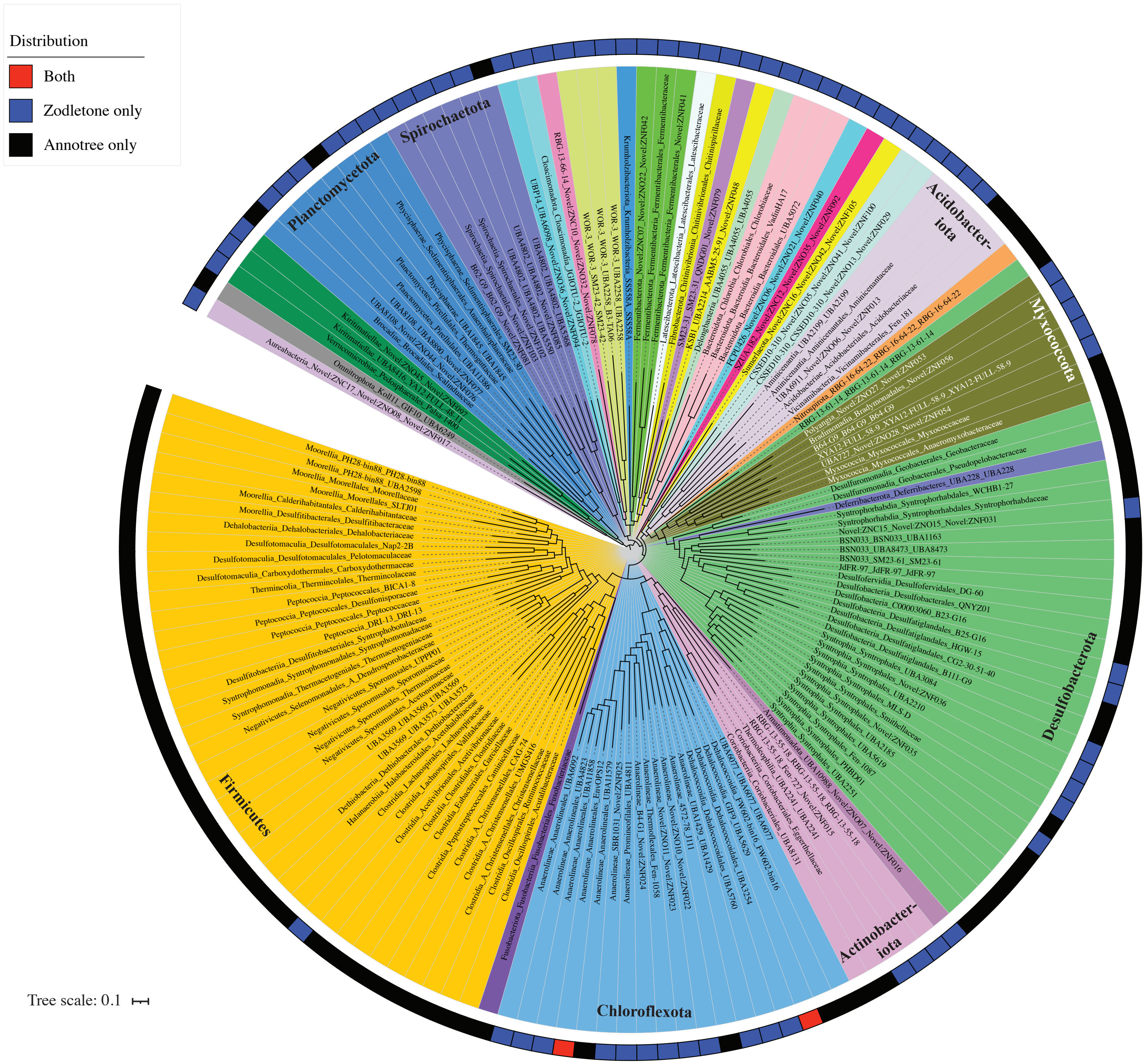
Distribution
Both
Zodletone only
Annotree only
Spirochaetota
Planctomycetota
Acidobacteriota
Myxococcota
Desulfobacterota
Actinobacteriota
Chloroflexota
Firmicutes
Tree scale: 0.1

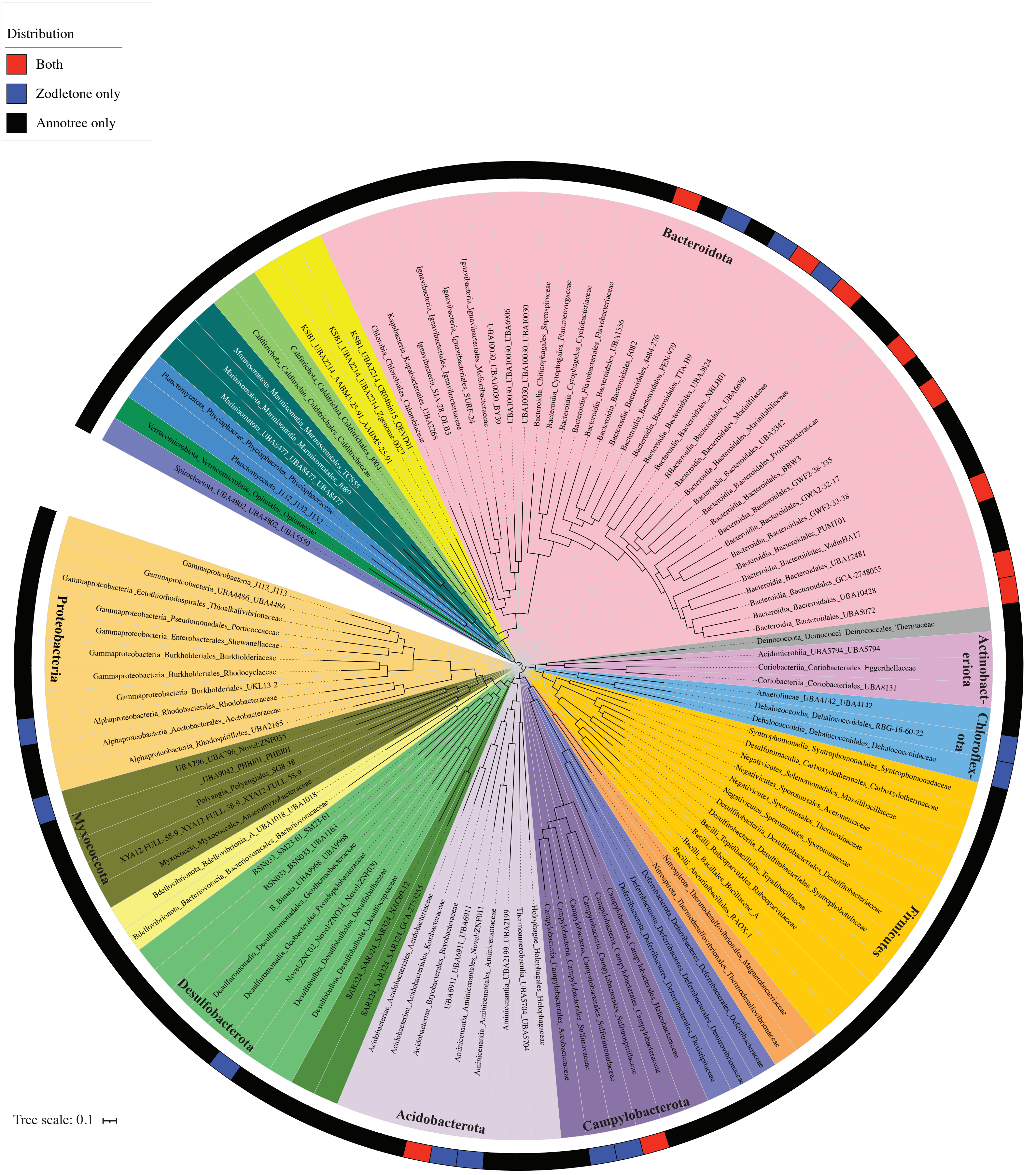
Distribution
Both
Zodletone only
Annotree only
Bacteroidota
Actinobacteriota
Chloroflexota
Firmicutes
Campylobacterota
Acidobacterota
Desulfobacterota
Myxococcota
Proteobacteria
Tree scale: 0.1
Bacteroidia_Bacteroidales_UBA5072
Bacteroidia_Bacteroidales_UBA10428
Bacteroidia_Bacteroidales_GCA-2748055
Bacteroidia_Bacteroidales_UBA12481
Bacteroidia_Bacteroidales_VadinHA17
Bacteroidia_Bacteroidales_PUMT01
Bacteroidia_Bacteroidales_GWF2-33-38
Bacteroidia_Bacteroidales_GWA2-32-17
Bacteroidia_Bacteroidales_GWF2-38-335
Bacteroidia_Bacteroidales_BBW3
Bacteroidia_Bacteroidales_Prolixibacteraceae
Bacteroidia_Bacteroidales_UBA5342
Bacteroidia_Bacteroidales_Marinilabiliaceae
Bacteroidia_Bacteroidales_Marinifilaceae
Bacteroidia_Bacteroidales_UBA6680
BBacteroidia_Bacteroidales_NBLH01
Bacteroidia_Bacteroidales_UBA3824
Bacteroidia_Bacteroidales_TTA-H9
Bacteroidia_Bacteroidales_FEN-979
Bacteroidia_Bacteroidales_4484-276
Bacteroidia_Bacteroidales_F082
Bacteroidia_Bacteroidales_UBA1556
Bacteroidia_Flavobacteriales_Flavobacteriaceae
Bacteroidia_Cytophagales_Cyclobacteriaceae
Bacteroidia_Cytophagales_Flammeovirgaceae
Bacteroidia_Chitinophagales_Saprospiraceae
UBA10030_UBA10030_UBA10030
UBA10030_UBA10030_UBA6906
UBA10030_UBA10030_BY39
Ignavibacteria_Ignavibacteriales_Melioribacteraceae
Ignavibacteria_Ignavibacteriales_SURF-24
Ignavibacteria_Ignavibacteriales_Ignavibacteriaceae
Ignavibacteria_SJA-28_OLB5
Kapabacteria_Kapabacteriales_UBA2268
Chlorobia_Chlorobiales_Chlorobiaceae
KSB1_UBA2214_CR04bin15_QEVD01
KSB1_UBA2214_UBA2214_Zgenome-0027
KSB1_UBA2214_AABM5-25-91_AABM5-25-91
Calditrichota_Calditrichia_Calditrichales_J004
Calditrichota_Calditrichia_Calditrichales_Calditrichaceae
Marinisomatota_Marinisomatia_Marinisomatales_TCS55
Marinisomatota_Marinisomatia_Marinisomatales_J089
Marinisomatota_UBA8477_UBA8477_UBA8477
Planctomycetota_Phycisphaerae_Phycisphaerales_Phycisphaeraceae
Planctomycetota_J132_J132_J132
Verrucomicrobiota_Verrucomicrobiae_Opitutales_Opitutaceae
Spirochaetota_UBA4802_UBA4802_UBA5550
Deinococcota_Deinococci_Deinococcales_Thermaceae
Acidimicrobiia_UBA5794_UBA5794
Coriobacteriia_Coriobacteriales_Eggerthellaceae
Coriobacteriia_Coriobacteriales_UBA8131
Anaerolineae_UBA4142_UBA4142
Dehalococcoidia_Dehalococcoidales_RBG-16-60-22
Dehalococcoidia_Dehalococcoidales_Dehalococcoidaceae
Syntrophomonadia_Syntrophomonadales_Syntrophomonadaceae
Desulfotomaculia_Carboxydothermales_Carboxydothermaceae
Negativicutes_Selenomonadales_Massilibacillaceae
Negativicutes_Sporomusales_Acetonemaceae
Negativicutes_Sporomusales_Thermosinaceae
Negativicutes_Sporomusales_Sporomusaceae
Desulfitobacteria_Desulfitobacteriales_Desulfitobacteriaceae
Desulfitobacteria_Desulfitobacteriales_Syntrophobotulaceae
Bacilli_Tepidibacillales_Tepidibacillaceae
Bacilli_Rubeoparvulales_Rubeoparvulaceae
Bacilli_Bacillales_Bacillaceae_A
Bacilli_Aneurinibacillales_RAOX-1
Nitrospirota_Thermodesulfovibrionales_Magnetobacteriaceae
Nitrospirota_Thermodesulfovibrionales_Thermodesulfovibrionaceae
Deferribacterota_Deferribacteres_Deferribacterales_Deferribacteraceae
Deferribacterota_Deferribacteres_Deferribacterales_Denitrovibrionaceae
Deferribacterota_Deferribacteres_Deferribacterales_Flexistipitaceae
Campylobacteria_Campylobacterales_Helicobacteraceae
Campylobacteria_Campylobacterales_Campylobacteraceae
Campylobacteria_Campylobacterales_Sulfurospirillaceae
Campylobacteria_Campylobacterales_Sulfurimonadaceae
Campylobacteria_Campylobacterales_Sulfurovaceae
Campylobacteria_Campylobacterales_Arcobacteraceae
Holophagae_Holophagales_Holophagaceae
Thermoanaerobaculia_UBA5704_UBA5704
Aminicenantia_UBA2199_UBA2199
Aminicenantia_Aminicenantales_Aminicenantaceae
Aminicenantia_Aminicenantales_Novel:ZNF011
UBA6911_UBA6911_UBA6911
Acidobacteriae_Bryobacterales_Bryobacteraceae
Acidobacteriae_Acidobacteriales_Koribacteraceae
Acidobacteriae_Acidobacteriales_Acidobacteriaceae
SAR324_SAR324_SAR324_GCA-2753255
SAR324_SAR324_SAR324_NAC60-12
Desulfobulbia_Desulfobulbales_Desulfocapsaceae
Desulfobulbia_Desulfobulbales_Desulfobulbaceae
Novel:ZNC02_Novel:ZNO14_Novel:ZNF030
Desulfuromonadia_Geobacterales_Pseudopelobacteraceae
Desulfuromonadia_Desulfuromonadales_Geothermobacteraceae
B_Binatia_UBA9968_UBA9968
BSN033_BSN033_UBA1163
BSN033_SM23-61_SM23-61
Bdellovibrionota_Bacteriovoracia_Bacteriovoracales_Bacteriovoracaceae
Bdellovibrionota_Bdellovibrionia_A_UBA1018_UBA1018
Myxococcia_Myxococcales_Anaeromyxobacteraceae
XYA12-FULL-58-9_XYA12-FULL-58-9_XYA12-FULL-58-9
_Polyangia_Polyangiales_SG8-38
_UBA9042_PHBI01_PHBI01
UBA796_UBA796_Novel:ZNF055
Alphaproteobacteria_Rhodospirillales_UBA2165
Alphaproteobacteria_Acetobacterales_Acetobacteraceae
Alphaproteobacteria_Rhodobacterales_Rhodobacteraceae
Gammaproteobacteria_Burkholderiales_UKL13-2
Gammaproteobacteria_Burkholderiales_Rhodocyclaceae
Gammaproteobacteria_Burkholderiales_Burkholderiaceae
Gammaproteobacteria_Enterobacterales_Shewanellaceae
Gammaproteobacteria_Pseudomonadales_Porticoccaceae
Gammaproteobacteria_Ectothiorhodospirales_Thioalkalivibrionaceae
Gammaproteobacteria_UBA4486_UBA4486
Gammaproteobacteria_J113_J113

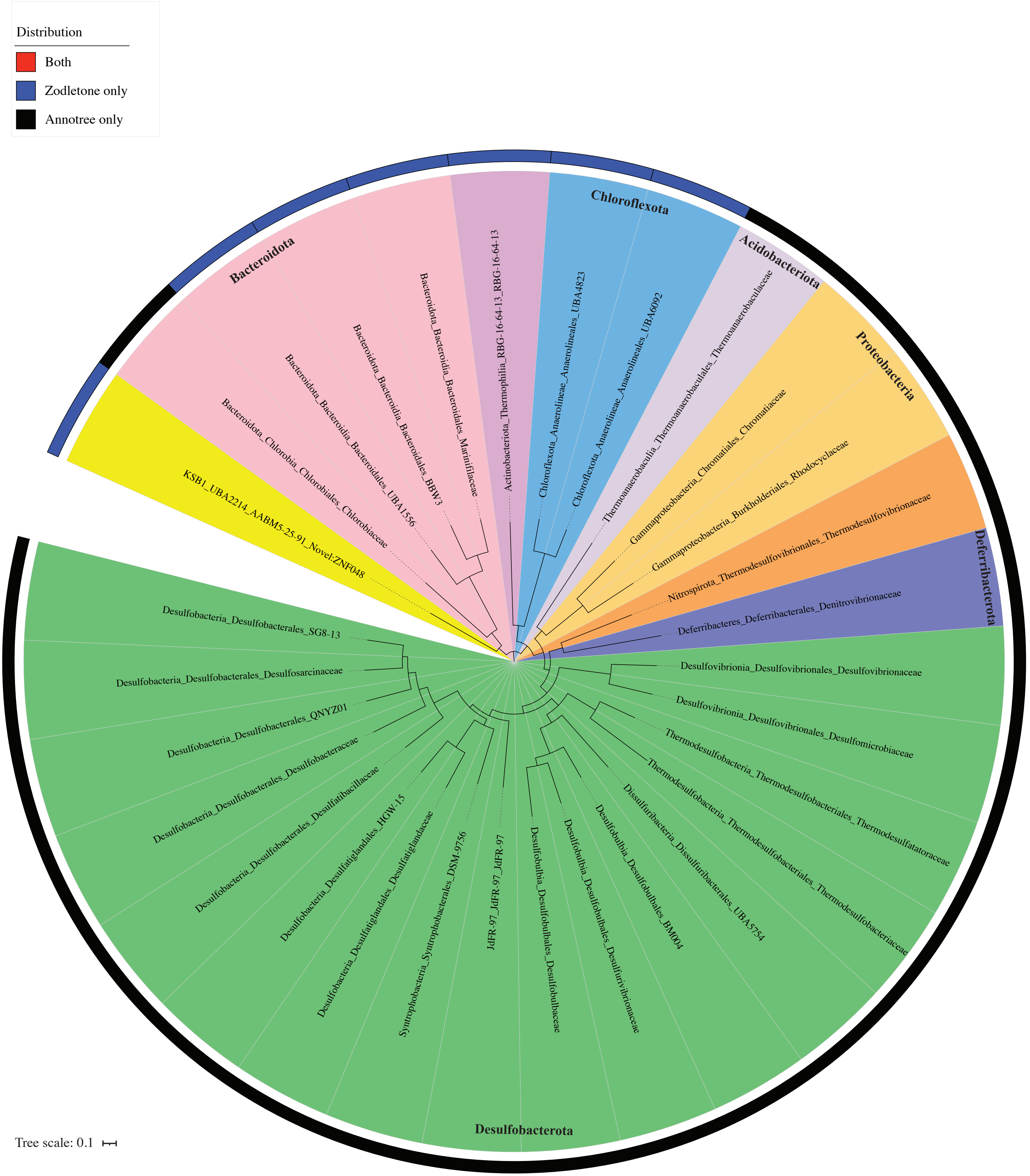

Distribution
Both
Zodletone only
Annotree only
Bacteroidota
Chloroflexota
Acidobacteriota
Proteobacteria
Deferribacterota
Desulfobacterota
KSB1_UBA2214_AABM5-25-91_Novel:ZNF048
Bacteroidota_Chlorobia_Chlorobiales_Chlorobiaceae
Bacteroidota_Bacteroidia_Bacteroidales_UBA1556
Bacteroidota_Bacteroidia_Bacteroidales_BBW3
Bacteroidota_Bacteroidia_Bacteroidales_Marinifilaceae
Actinobacteriota_Thermophilia_RBG-16-64-13_RBG-16-64-13
Chloroflexota_Anaerolineae_Anaerolineales_UBA4823
Chloroflexota_Anaerolineae_Anaerolineales_UBA6092
Thermoanaerobaculia_Thermoanaerobaculales_Thermoanaerobaculaceae
Gammaproteobacteria_Chromatiales_Chromatiaceae
Gammaproteobacteria_Burkholderiales_Rhodocyclaceae
Nitrospirota_Thermodesulfovibrionales_Thermodesulfovibrionaceae
Deferribacteres_Deferribacterales_Denitrovibrionaceae
Desulfovibrionia_Desulfovibrionales_Desulfovibrionaceae
Desulfovibrionia_Desulfovibrionales_Desulfomicrobiaceae
Thermodesulfobacteria_Thermodesulfobacteriales_Thermodesulfatatoraceae
Thermodesulfobacteria_Thermodesulfobacteriales_Thermodesulfobacteriaceae
Dissulfuribacteria_Dissulfuribacterales_UBA5754
Desulfobulbia_Desulfobulbales_BM004
Desulfobulbia_Desulfobulbales_Desulfurivibrionaceae
Desulfobulbia_Desulfobulbales_Desulfobulbaceae
JdFR-97_JdFR-97_JdFR-97
Syntrophobacteria_Syntrophobacterales_DSM-9756
Desulfobacteria_Desulfatiglandales_Desulfatiglandaceae
Desulfobacteria_Desulfatiglandales_HGW-15
Desulfobacteria_Desulfobacterales_Desulfatibacillaceae
Desulfobacteria_Desulfobacterales_Desulfobacteraceae
Desulfobacteria_Desulfobacterales_QNYZ01
Desulfobacteria_Desulfobacterales_Desulfosarcinaceae
Desulfobacteria_Desulfobacterales_SG8-13
Tree scale: 0.1

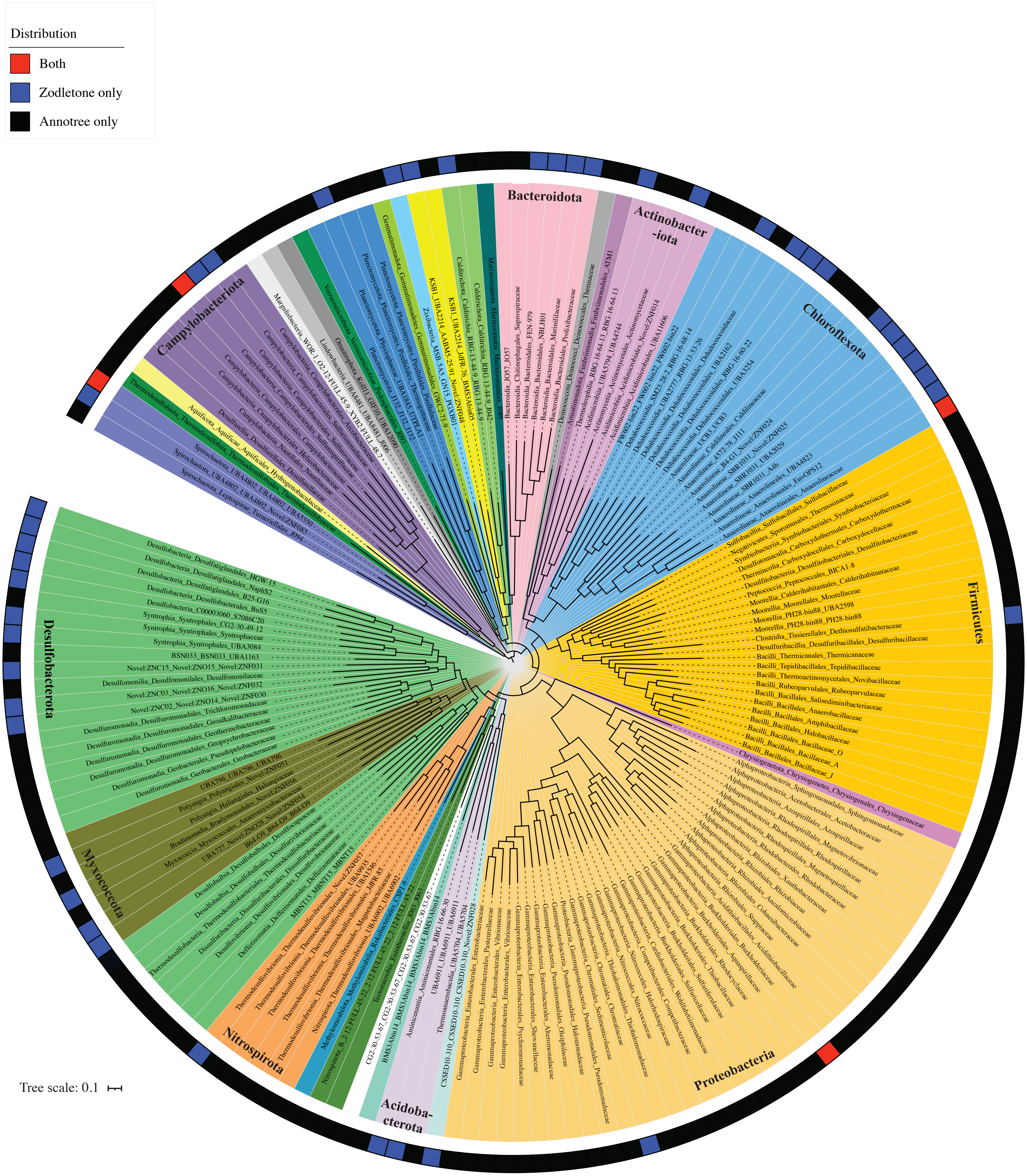
Distribution
Both
Zodletone only
Annotree only
Bacteroidota
Actinobacter-iota
Chloroflexota
Firmicutes
Proteobacteria
Acidoba-cterota
Nitrospirota
Myxococcota
Desulfobacterota
Campylobacteriota
Tree scale: 0.1
